# Hydroseismograms at Gran Sasso aquifer, central Italy, for earthquake hydrology studies

**DOI:** 10.1038/s41598-025-96113-4

**Published:** 2025-04-16

**Authors:** Domenico Isaya, Gaetano De Luca, Giuseppe Di Carlo, Vincenzo Guerriero, Raffaele Martorana, Marco Tallini

**Affiliations:** 1 Department of Civil, Construction-Architectural and Environmental Engineering, University of L’Aquila, via Giovanni Gronchi 18, L’Aquila, 67100 Italy; 2 National Institute of Geophysics and Volcanology (INGV), L’Aquila, Italy; 3 Gran Sasso National Laboratories, INFN, Assergi (L’Aquila), L’Aquila, Italy; 4 Department of Earth and Marine Sciences (DiSTeM), University of Palermo, Palermo, Italy

**Keywords:** Earthquake hydrology, Hydroseismogram, Hydrosensitive site to seismicity, Hydraulic pressure device, Carbonate aquifer, Central Italy, Geophysics, Hydrogeology, Seismology, Hydrology, Natural hazards, Solid Earth sciences, Techniques and instrumentation

## Abstract

The use of hydroseimograms (i.e., high frequency pore pressure monitoring in wells in rock) emerges as a promising tool for a better understanding of the physics underlying earthquakes, as well as for seismic activity monitoring and/or prediction. This study investigates the earthquake detection capability of a hydraulic pressure device (HPD) installed in the Gran Sasso aquifer (GSA), Italy. The HPD, located in boreholes S13 and S14, near the INGV seismic station, GIGS, monitors hydraulic pressure changes within the aquifer. We compared data from the HPD and from GIGS to assess the HPD’s ability to detect earthquakes. The analysis covered the period from May 2015 to December 2023. The HPD successfully identified 148 out of 1068 analyzed earthquakes. Compared to previous studies, our HPD displayed significantly higher sensitivity, particularly for crustal earthquakes. These HPD devices show distinctive features, such as deep location, high frequency sampling (20 Hz), and hosting wells intercepting the main fault network. The unique location of the HPD, combined with its sensitivity to seismic events, makes it a valuable tool for continuous monitoring of earthquake activity, coupled with pore pressure trends and anomalies, in the region. These results confirm the potential of the HPD system for earthquake detection within the GSA. Future studies will continue to evaluate the HPD’s capabilities and its role in earthquake hazard assessment, as well as its potential use in other areas worldwide.

**Supplementary Information:**

The online version contains supplementary material available at 10.1038/s41598-025-96113-4.

## Introduction

**Fig. 1:**
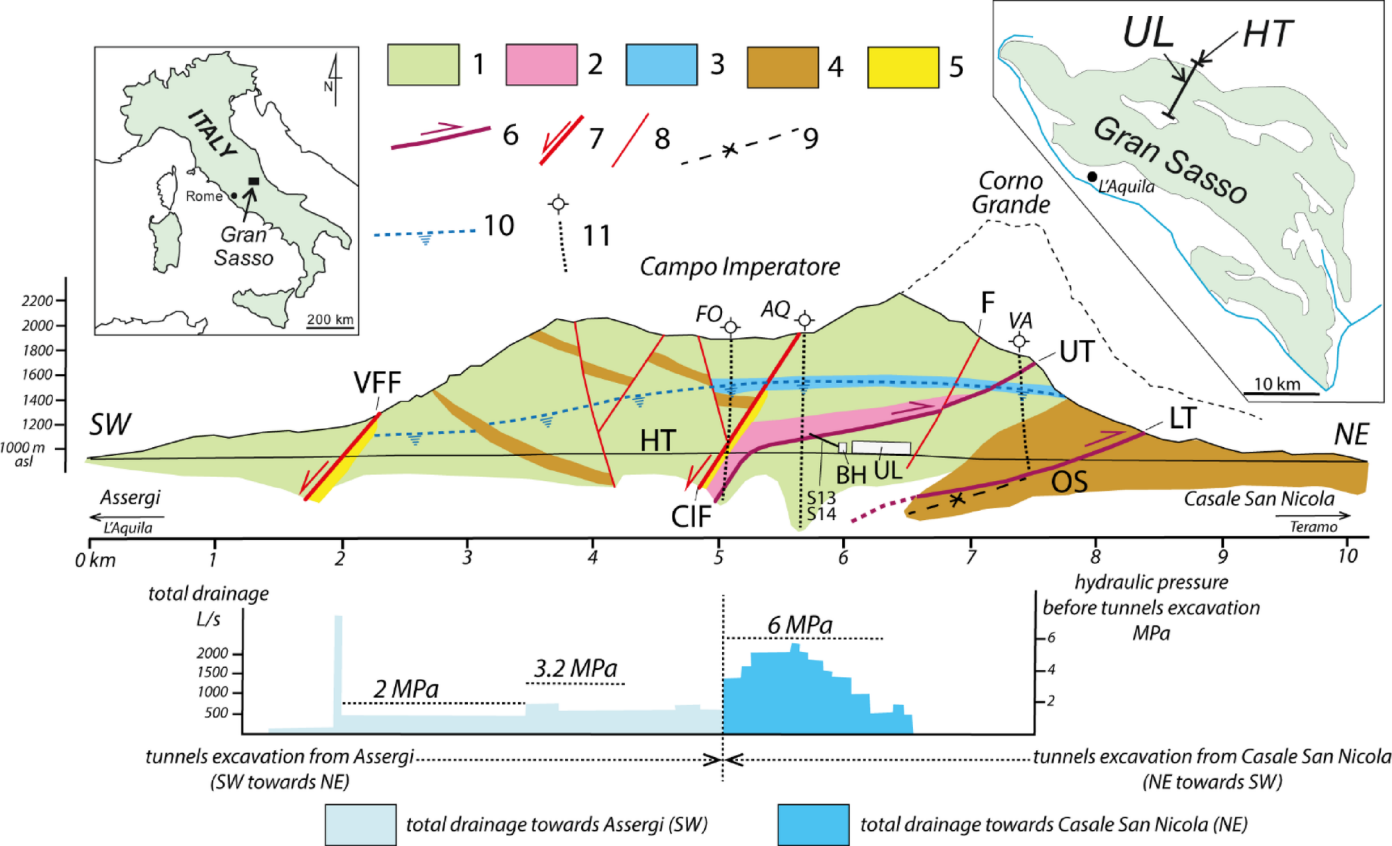
Top: GSA hydrogeological section including the highway tunnels (HT), the Gran Sasso INFN underground laboratory (UL), the borehole hall (BH) and the area of S13-S14 subhorizontal boreholes. 1- limestone (Upper Cretaceous - Upper Jurassic); 2- dolomite (Dolomia Principale Fm., Upper Triassic); 3- paleokarst horizon; 4- terrigenous low-permeability lithologies; 5- low-permeability fault rock; 6- main thrust (UT: upper thrust; LT: lower thrust); 7- main normal fault (CIF: Campo Imperatore fault; VFF: Valle Fredda fault); 8- minor normal fault (F behaves hydraulically as a drain); 9- Overturned syncline (OS) in the UT footwall; 10- water table before the tunnels excavation; 11- deep boreholes (FO: Fontari; AQ: Monte Aquila; VA: Vaduccio). Bottom: groundwater drainage during tunnels excavation (left Y-axis) and hydraulic pressure before tunnels excavation (right Y-axis); HT- highway tunnel; BH- borehole hall; UL- Gran Sasso underground laboratory; S13 S14- subhorizontal boreholes. (redrawn from^27,29,34^).

**Fig. 2:**
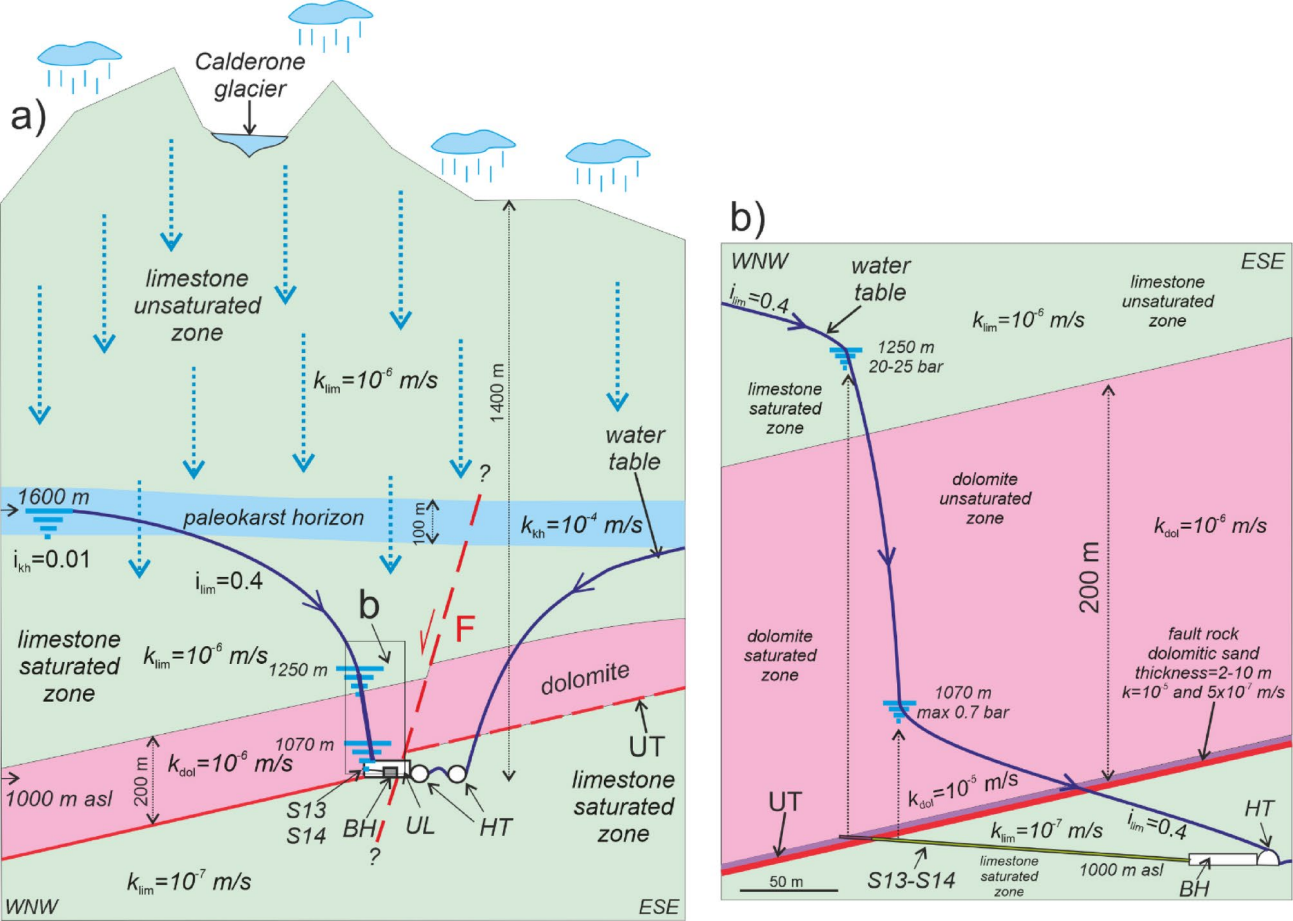
(**a**) GSA scheme transversal to the highway tunnels (HT) and passing through the Gran Sasso INFN underground laboratory (UL), borehole hall (BH) and S13-S14 area. (redrawn from De Luca et al., 2018). Calderone glacier represents the high elevation water reservoir – preferential recharge area) of the GSA; The hydrogeological setting in the square is shown into details in (**b**). The Calderone glacier acting as a water reservoir for the carbonate aquifer down below; i: hydraulic gradient; k: hydraulic conductivity (kh: paleokarst horizon; lim: limestone; dol: dolomite). The hydrogeological setting in the square is shown in detail in (**b**).

**Fig. 3:**
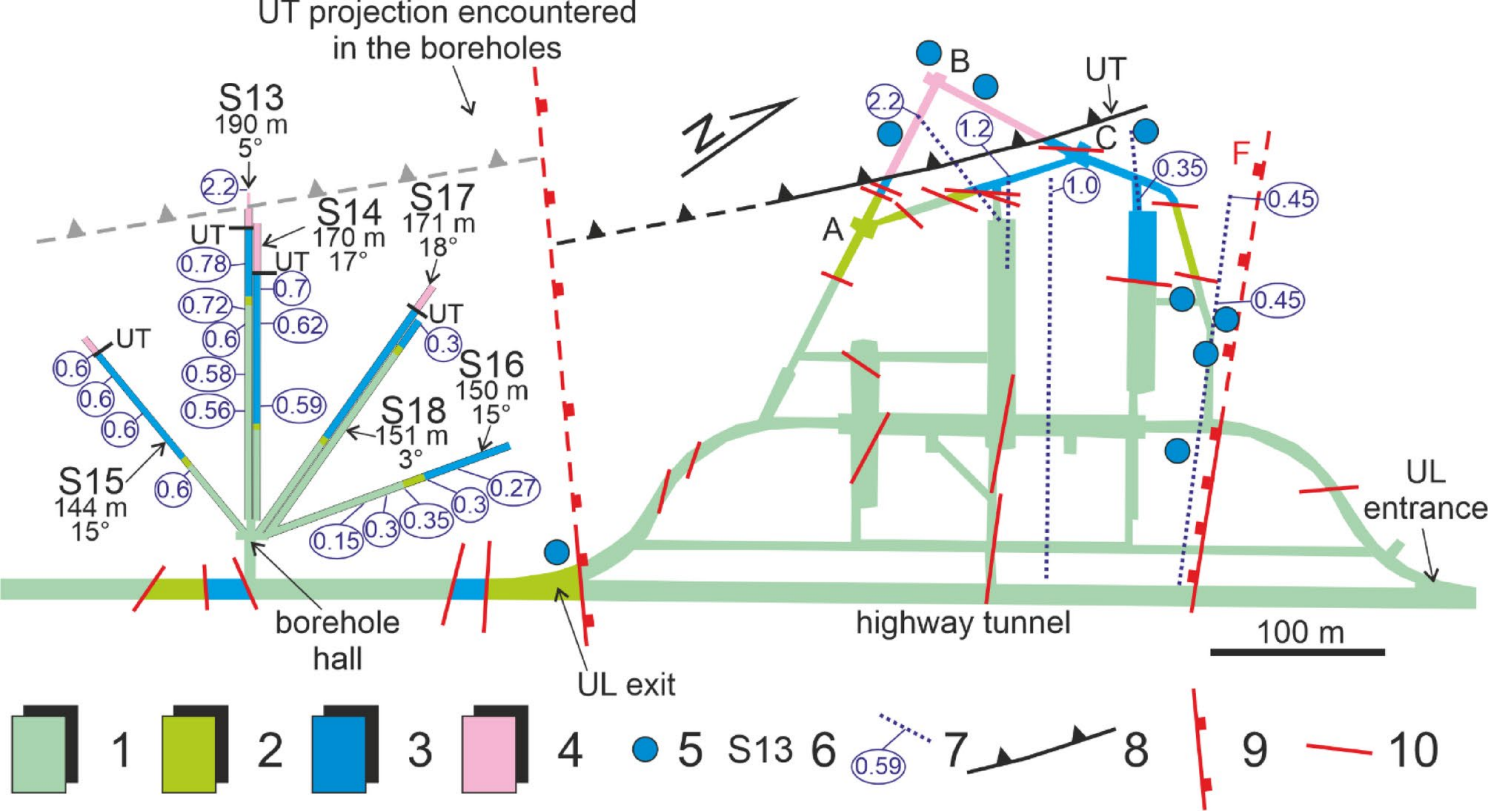
Geological map of underground laboratories (UL) at right side, and borehole hall at left side. The scheme shows the characteristics of the 6 horizontal boreholes (S13, S14, S15, S16, S17 and S18). The S13 and S14 boreholes were monitored in this study. (1) stratified limestone (Upper Cretaceous); (2) detrital massive limestone with local marly intercalations (middle Cretaceous); (3) stratified limestone with cherty layers (Lower Cretaceous - Upper Jurassic); (4) stratified dolomite (Upper Triassic); the dolomite (4) close to the thrust UT, belonging to its fault damage zone, is strongly tectonized; (5) remarkable concentrated groundwater flow; (6) borehole with whole length (m) and dip angle (°); (7) hydraulic pressure (MPa) measured during borehole drilling; (8) upper thrust (UT), dashed if inferred; (9) minor normal fault, dashed if inferred; (10) fault. UL: underground laboratory; A, B and C refer to the nodes of the two interferometers^42^. (redrawn from^2,29^).

High-frequency (10–50 Hz) monitoring of hydraulic pressure, electrical conductivity, and temperature within wells in rock, coupled with seismic data analysis, presents a promising avenue for elucidating seismic related and wave propagation mechanics. Previous research conducted by De Luca et al.^1,2^ highlighted anomalies in the signals recorded in horizontal wells within a carbonate aquifer (S13 and S14 boreholes, Gran Sasso Tunnel, Italy, Figs. 1, 2 and 3), in pre-, co- and post-seismic condition, during the 2016 Amatrice seismic sequence, underscoring the potential of these monitoring techniques.

Hydrologic responses to earthquakes include post-seismic long-term effects, such as changes in stream and spring discharge, in the water level in wells, etc., as well as co-seismic high-frequency pore pressure changes in wells. Over the last decades, these effects have been detected and measured in several sites worldwide, in wells showing epicentral distance spanning from zero to thousands of kilometers^3–15^.

Although this study focuses on the hydrological response to nearby or distant seismic events rather than the effect of hydrological processes on earthquake triggering, we point out that these two topics share common ground. Recent studies have highlighted the potential influence of fracture and crack network attributes, as well as ground fluid flow, on earthquake initiation^16–18^. Moreover, even in low-porosity carbonate rocks, small-scale joints and microfractures are ubiquitous and pervasive within the rock mass^19^. Therefore, we speculate that a deeper understanding of the hydrological effects of seismic events can also contribute to clarifying seismicity induced by hydrological phenomena.

The present investigation delves into long-term high frequency pore pressure data, compared with seismic records from proximate stations, to enhance our comprehension of these phenomena.

The Gran Sasso Aquifer (GSA), characterized by its fractured-karst geology, multiple active faults, and location within Italy’s high seismic hazard zone, serves as an exemplary case study. Moreover, the presence of the Italian Institute of Nuclear Physics (INFN) underground laboratory (UL) within the GSA provides a unique opportunity to study aquifer-earthquake interactions in a deep saturated environment largely unaffected by shallow hydrological processes.

As a representative Mediterranean carbonate aquifer, the Gran Sasso has undergone extensive investigation also thanks to the excavation of significant underground infrastructure, including two 10 km long highway tunnels crossing transversally the whole GSA (HT) and the UL. Initially, the discharges of many GSA springs declined due to tunnels excavation, however, the discharge values subsequently stabilized. Since the 1980s, after tunnels excavation, groundwater from the GSA core is drained. It is captured for drinking purposes through two drainage channels located below HT feeding the groundwater intake plant (Figs. 1 and 2). Decades of hydrogeological studies and the execution of deep boreholes have yielded substantial data which have allowed to formulate a hydrogeological model^20–41^.

The UL has been instrumental in monitoring groundwater parameters during and after the 2009 L’Aquila and 2016–2017 Central Italy seismic sequences^1,2,22,37^. Also, during these sequences, groundwater flow rate anomalies in the GSA aquifer were detected in pre-, co- and post-seismic condition^38^. Building upon these foundational studies, this research evaluates the efficacy of a high-frequency hydraulic pressure device (HPD) installed in boreholes S13 and S14 (Figs. 1, 2 and 3) for earthquake detection. By comparing HPD data with records from the National Institute of Geophysics and Volcanology (INGV) seismic station GIGS within the UL, we aim to assess the HPD’s sensitivity to seismic events.

This comprehensive analysis will contribute to a better understanding of the complex interplay between groundwater systems and seismic activity, ultimately advancing our ability to monitor and mitigate earthquake-related hazards.

## Hydrogeological and structural setting of Gran Sasso aquifer

The Gran Sasso aquifer (hereafter GSA), a 1000 km² wide carbonate formation, is characterized by fissured, fault-partitioned, and partially karstified volumes within its vadose zone. The aquifer’s core hosts the LNGS-INFN underground laboratories (UL) and the borehole hall containing several hundred-meter-long horizontal boreholes, including the monitored S13 and S14 (Figs. 1, 2 and 3). The GSA, a representative Apennine carbonate aquifer, has been extensively studied due to the construction of two 10-km-long highway tunnels traversing it, and the establishment of INFN underground laboratories. Decades of hydrogeological investigations and deep borehole drilling have provided substantial data for developing detailed hydrogeological models^21–25,27–29,31–41^.

GSA lithology comprises Meso-Cenozoic basin-to-slope and reef-platform carbonate rocks, structured within a contractional thrust belt formed during the upper Miocene Apennines orogeny (in Fig. 1, UT, LT and OS). Subsequent SW-dipping Plio-Quaternary extensional faults (in Fig. 1 CIF and VFF), associated with the NE-shifting Tyrrhenian post-orogenic extensional front, further influenced the aquifer’s structural framework^43^.

**Fig. 4:**
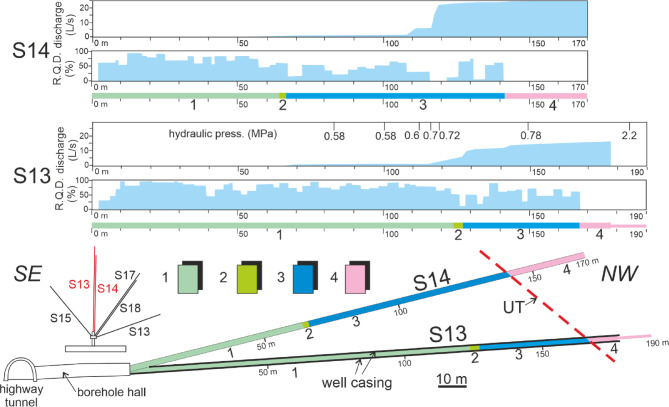
Top: geological logs of S13 and S14 boreholes (Figs. 1 and 2 for their location); the hydraulic pressure (MPa) and the groundwater discharge measured during the boring are reported. bottom: geological section and map location; the black bold lines of S13 borehole represent the iron tube (thickness: 150 mm). (1) stratified limestone (Scaglia Fm.) Upper Cretaceous; (2) detrital massive limestone with local marly intercalations (detrital Fucoidi Fm.) middle Cretaceous; (3) stratified limestone with cherty layers (Terratta and Limestone with radiolarians Fm.) Lower Cretaceous - Upper Jurassic; (4) stratified dolomite (Dolomia Principale Fm.) Upper Triassic (aquitard); UT: upper thrust. (redrawn from^2,29^).

In the area of HT and UL, GSA is hydraulically confined to the north and east by Miocene terrigenous aquicludes (Figs. 1 and 2). Locally, in the LT hangingwall, the UL and borehole area intersect the local upper thrust (UT in Figs. 1, 2, 3 and 4). The Lower Liassic limestones - Upper Triassic dolostones, as hangingwall unit, overlay along UT onto upper Cretaceous-upper Jurassic mud-supported detrital and cherty limestones, as the footwall unit. These last lithologies represent the SW-dipping flank of an overturned syncline whose core and normal flank are composed by Miocene terrigenous lithologies. Within the normal flank of the syncline the regional SW-dipping lower thrust is located. So that the permeability boundary is represented by the carbonate rocks of the overturned flank laying onto the Miocene terrigenous lithologies of the syncline core. The volume constituted by the carbonate rocks superimposed on the terrigenous ones is a compartmentalized part of the GSA aquifer. This is bounded to the NE by the above-mentioned low permeability boundary, while to the SW by the low permeability fault rock (k = 10^− 7^ m/s, *n* = 10%, where k and n refer to hydraulic permeability and porosity respectively, also hereafter) of the extensional and seismogenic Campo Imperatore fault (CIF in Fig. 1) dipping to the SW at 60°.

In the GS vadose zone groundwater flow is affected by the epikarst, above all located below the endorheic basins like Campo Imperatore and the significative 100 m-thick paleokarst horizon located between 1700 and 1600 m asl between Campo Imperatore, to the SW, and the NE GS slope (k = 10^− 4^ m/s; *n* = 30–50%). Significant groundwater circulation occurs in the vadose zone through this paleokarst horizon (Figs. 1 and 2).

In the GS phreatic zone, dual groundwater flow velocity has been hypothesized based on hydrochemical isotope hydrological data^40^, according to the hydraulic conductivity values estimated for the several NW-SE oriented faults (k = 10^− 4^ m/s) and for fracture rock mass network (k = 10^− 6^÷10^− 7^ m/s; *n* = 10%)^24^ (Figs. 1 and 2).

Also, interferometric data compared to groundwater discharge data allows to fine tune the groundwater flow velocity according to a dual porosity model with an anisotropic behaviour of hydraulic conductivity^25^. The ground deformation was measured by two paired 90 m long interferometers (BA and BC), located in the UL area^42^ (Fig. 3). Changes in deformation was correlated with the seasonal recharge/discharge hydrogeological cycle by comparing longitudinal strain measured in the two perpendicular directions (NW-SE and NE-SW interferometers corresponding to A-B and BC respectively in Fig. 3) with the discharges of springs fed by part of GSA located in the UL and HT aquifer volume^25^.

Regarding the discontinuities of the rock mass and their orientation with respect to the two interferometers, a prevalent family of NE-SW oriented small-scale joints can be recognized which orthogonally crosses the NE-SW interferometer^25^. Conversely, the NW-SE oriented fault F is perpendicular to the NE-SW interferometer. The hydraulic conductivity of the predominantly NE-SW oriented fractures crossing pervasively the rock mass is lower (k = 10^− 6^÷10^− 7^ m/s) than that of the NW-SE oriented extensional faults (k = 10^− 4^ m/s) (e.g., F crossing the entrance of UL in Figs. 1, 2 and 3)^24^.

During aquifer recharge, the groundwater table rises. In this phase, the NW-SE interferometer (A-B in Fig. 3) shows a contraction, while the NE-SW oriented one (B-C in Fig. 3) exhibits dilation. The contraction in the NW-SE direction is explained by the absence/delay of pore pressure increase, despite a higher total pressure, due to the very low hydraulic conductivity along the NE-SW oriented joints. Conversely, the dilation in the NE-SW direction is explained by the faster increase in water pressure along fault F, which exhibits high hydraulic conductivity values^25^. The opposite case occurs during the discharge phase when the water table is lowered. This behavior can be explained through the fracture permeability model for rocks illustrated by Guerriero et al.^44,45^, where hierarchical fracture systems, under non-steady state fluid flow conditions, exhibit significantly different response times. During fluctuations in the water table level, the different permeability between hierarchical fracture systems (e.g., pore scale system, small joints, fault networks, etc.) causes a heterogeneous pore pressure field. For instance, during a rise of water table, the resulting pressure increase occurs significantly slower in small-scale joints compared to fault networks. Consequently, in these joints, the total pressure increase is not counterbalanced by the increase in pore pressure^46,47^, leading to a reduction in their aperture. Conversely, the more rapid increase in pore pressure within the more permeable faults can cause an increase in their volume.

At local scale, in the UL and BH area, the Upper Triassic dolomites located in the hangingwall just above the UT plane are low permeable (k = 10^− 6/−7^ m/s; *n* = 2-5%) except in some fractured and faulted zones (k = 10^− 5^ m/s; *n* = 20%) as demonstrated by (i) the low RQD values ​​and (ii) the significant drained discharge rates measured in these lithologies and (iii) by the notable hydraulic pressure values ​​measured (Figs. 3 and 4). More precisely, hydraulic pressure within the 150–200 m-long horizontal boreholes of BH typically ranges from 0.5 to 0.7 MPa (50–70 m head), except for S13, which exhibits significantly higher pressures of 2.0-2.5 MPa (200–250 m head) (Fig. 4). The 2–10 m thick UT fault rock behaves locally as a drain (k = 10^− 5^ m/s; porosity = 35%) and sometimes as a low-permeability septum (k = 5 × 10^− 7^ m/s). The UT footwall limestones are again low permeable (k = 10^− 7^ m/s; *n* < 5%) and show little groundwater inflow apart from the fault F (Figs. 3). This last appears to be a notable drain as showed by the significant groundwater discharge here measured (Fig. 3).

## Materials and methods

### The water pressure device (HPD) and the INGV seismic station GIGS

The seismic station GIGS (https://terremoti.ingv.it/en/instruments/station/GIGS) is part of the Italian Seismic Network (https://terremoti.ingv.it/en/instruments/network/IV) and is managed by INGV. It is used for both local and regional seismic monitoring, as well as for recording global seismicity. The HPD, on the other hand, serves different purposes, as it is designed for continuous measurements of hydraulic pressure and pore pressure phenomena within the aquifer, including slow and high-frequency variations resulting from various natural causes, such as earthquakes, climatic phenomena, earth tides, and seasonal variations. Since the HPD performs high-frequency measurements, it is also suitable for providing hydroseismograms. These can be useful for many purposes, such as additional monitoring of seismic activity, but also to identify pre-, post- and co-seismic anomalies of pore pressure in the rock mass, as well as anomalies and precursors in the seismic traces that are not identifiable by standard seismic stations^2^.

The Gran Sasso carbonate aquifer hosts the Gran Sasso National Laboratories (LNGS-INFN), in the vicinity of which six horizontal boreholes are located within the borehole hall, denoted by S13, S14, S15, S16, S17, and S18 (Figs. 2 and 3).

Drilled in the late 1980s and early 1990s, these boreholes were conceived as exploratory investigations to support the potential expansion of the INFN’s national laboratories. Their primary purpose was to provide valuable information on water pressures at the site for future excavation planning. However, the expansion project was never fully realized, and consequently, the borehole chamber remained available for ongoing aquifer studies by INGV researchers.

Figures 2 and 3 graphically illustrate the arrangement of these wells within the borehole hall. For this study, the focus was on the adjacent boreholes S13 and S14. Borehole S13, drilled in the late 1980s, has a horizontal length of 190 m and gently slopes upward by approximately 5 degrees. It intersects a fault near its end. The first 175 m of borehole S13 are cased, while the remaining 10 m drain into the Upper Triassic dolomite. Additionally, it is located approximately 39 km southeast of the epicenter of the 2016 Amatrice earthquake (6.0 Mw) and about 20 km northeast of the epicenter of the 2009 L’Aquila earthquake (6.3 Mw).

Borehole S14, with a length of 170 m, drains into the same rock formation as borehole S13.

This apparatus differs from most of those used in the literature^3–15^ as it is located at a depth of more than one kilometer, below the water table, within horizontal boreholes subject to a strong hydraulic gradient, and performs high-frequency pore pressure measurements (20 Hz). Additionally, boreholes S13 and S14 intersect a major structure (UT) belonging to the fault network of the area (Fig. 3).

Within the Gran Sasso underground laboratories (LNGS-INFN), approximately 250 m from borehole S13, lies the INGV seismic station GIGS^2^. This station, part of the GINGER experiment^48^, is equipped with two broadband seismometers, enabling the continuous monitoring of microseismic activity in the Gran Sasso range and the recording of global seismicity.

### Data from HPD and GIGS

The Borehole Hall (BH), situated near the UL at an elevation of 965 m above sea level (asl), houses six horizontal boreholes, including the monitored S13 and S14. Approximately 250 m away, the UL hosts the INGV seismic station GIGS. For this study, we focused on the adjacent boreholes S13 and S14.

Borehole S13 is a 190-meter horizontal well with a gentle upward slope of around 5 degrees. It intersects a fault near its end (UT in Figs. 2 and 3). The first 175 m are encased, while the last 10 m drain within the Upper Triassic dolomite (Fig. 1). Borehole S14, with a similar length of 170 m, also drains within the same rock formation.

**Fig. 5:**
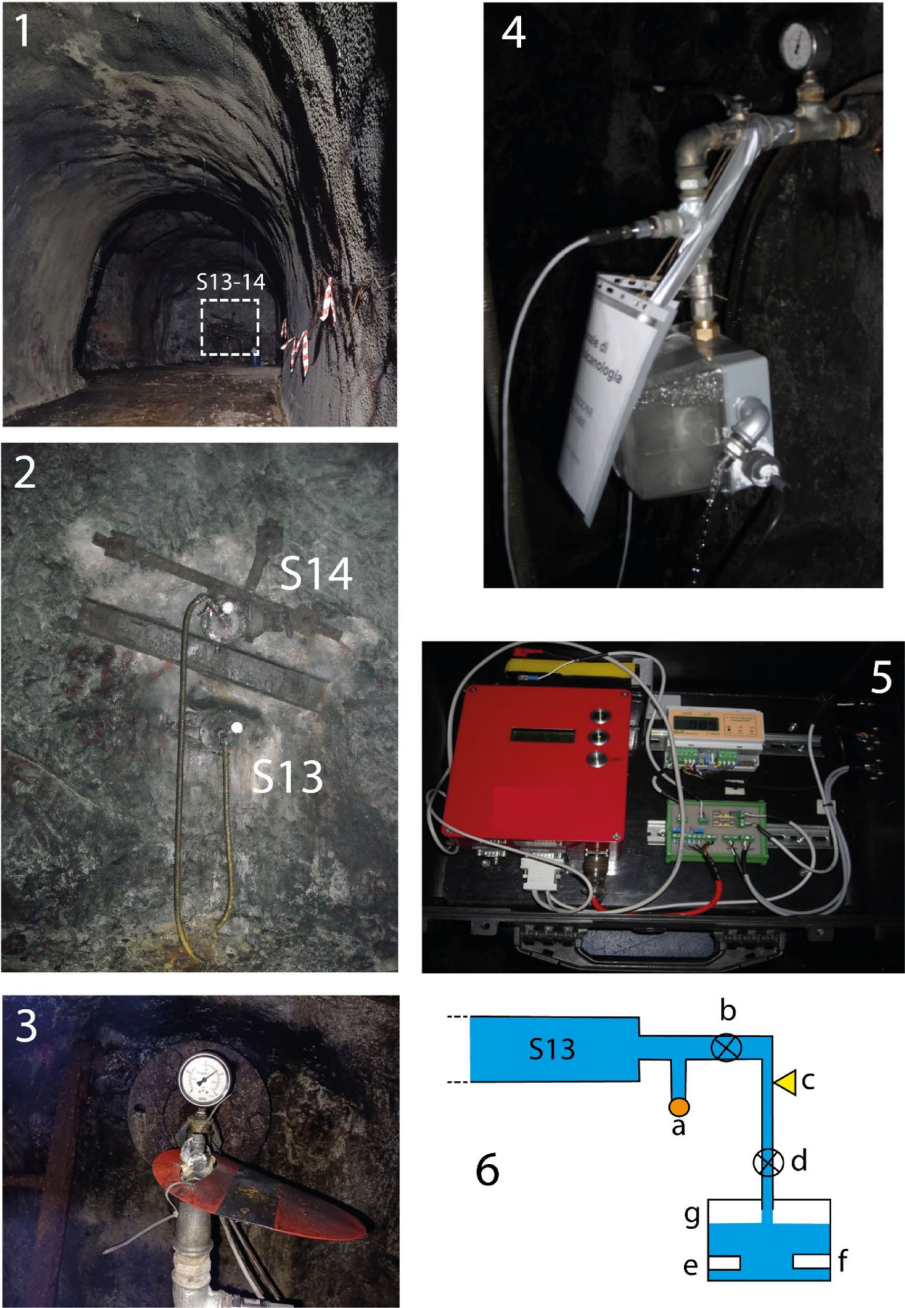
1: borehole hall with the location of the boreholes S13 and S14; 2: the boreholes S13 and S14; 3: the old analogic manometer of borehole S13; 4: Details of the hydraulic apparatus device (S14); 5: Analog-to-digital converter (model ADC SL06 by SARA, https://www.sara.pg.it/) with three 24 bit channels and acquisition frequency until 50 Hz for each channel; 6: hydraulic scheme of data acquisition apparatus (not in scale). S13 horizontal borehole length: 190 m. (a) old analogic manometer; (b) hydraulic valve, always open during the data acquisition; (c) sensor for hydraulic pressure; (d) hydraulic valve not completely close to enable the measurement of temperature and electrical conductivity in the box (g); (e) temperature sensor; (f) electrical conductivity sensor; (g) transparent plastic container housing the temperature and electrical conductivity sensors; water is expelled when reaching about 75% of the container volume.

Both S13 and S14 were equipped with a high-frequency (20 Hz) data acquisition system specifically designed for sensitive hydraulic pressure monitoring. This system utilizes a 3-channel, 24-bit analog-to-digital converter (ADC) model SL06 by SARA Electronic Instrument company (http://www.sara.pg.it/). The hydraulic scheme of the experimental apparatus (Fig. 5) is composed by (a) the horizontal boreholes; (b) an old analogic manometer; (c) a hydraulic valve always open during the data acquisition periods; (d) hydraulic pressure sensor; (e) hydraulic valve not completely close to enable the measurement of temperature and electrical conductivity in a container (h); (f) temperature sensor; (g) electrical conductivity sensor; (h) transparent plastic container housing the temperature and electrical conductivity sensors. Water is expelled when reaching about three quarters of the volume of the container.

Hydraulic pressure measurements at the heads of the 150–200 m boreholes typically ranged from 0.5 to 0.7 MPa (piezometric head of 50–70 m). However, borehole S13 exhibited a significantly higher pressure of 2.0-2.5 MPa (piezometric head of 200–250 m), suggesting a potential connection to the nearby fault zone.

Within the UL, the INGV seismic station GIGS is equipped with two broadband seismometers (Nanometrics Trillium 240s and Guralp CMG 3T 360s). This station plays a dual role: contributing to the GINGER experiment for rotational seismology^48,49^ and serving as a key observatory for monitoring seismic activity. The station is equipped to capture both continuous microseismic tremors within the Gran Sasso Aquifer (GSA) and seismic events on a broader scale, including regional Italian seismicity and teleseisms (distant earthquakes).

The primary objective of the joint analysis of well and seismic data consists in identifying and correlating coincidences between earthquakes of various scales (global, regional, and local) with the hydraulic pressure variations within the GSA detected by the HPDs installed in boreholes S13 and S14. This correlation analysis has the potential to improve our understanding of the interplay between seismic activity and groundwater dynamics. By comparing hydroseismograms generated from the HPD data and the seismograms recorded by the GIGS station, we aim to establish whether pressure fluctuations in the boreholes can be directly linked to specific seismic events.

The data acquisition, observation, and analysis period spanned from May 1st, 2015, to December 31st, 2023, with ongoing monitoring continuing. This extended timeframe allows for a comprehensive assessment of potential correlations between seismic events and hydraulic pressure variations within the GSA. The ongoing monitoring efforts will further contribute to this analysis and potentially lead to new insights into earthquake-induced hydrological phenomena.

### Data acquisition criteria and the utilized software in data processing

Data acquisition was conducted based on the researchers’ experience and, following the approach of Manga and Wang^13^, we searched for coincidences between global seismicity and hydraulic pressure signals recorded by the device at the head of the horizontal boreholes S13 and S14 (coordinates: 42.449883° 13.572717°).

Drawing on previous studies in the same aquifer, we established specific criteria to exclude events undetectable by the aquifer under investigation. These criteria linked a range of magnitudes to a range of distances from boreholes S13 and S14. To identify seismic events meeting these criteria, we queried the Italian National Institute of Geophysics and Volcanology’s website (http://terremoti.ingv.it) using the “custom search” function.

A database was created, chronologically listing events regardless of magnitude or distance. Acquired seismic data included: event number, date, time (Italian and international), magnitude, zone, depth, latitude, longitude, epicentral distance, azimuth, signal duration, maximum and minimum peaks, peak-to-peak, and bar conversion.

Particular attention was paid to the azimuth, as it provides crucial information about the wave type. The azimuth is the angle measured clockwise from geographic north to the Gran Sasso aquifer and the earthquake epicenter.

Subsequently, data from the ADC connected to boreholes S13 and S14 corresponding to the events of interest were provided. The resulting hydroseismograms were in miniseed format, with 6-hour files starting at midnight.

Seismic data from the national network station GIGS (https://terremoti.ingv.it/instruments/station/GIGS) were also downloaded in miniSEED format using a query string. This process involved modifying dates, times, and selecting the desired components (HH for all, HHZ for vertical, HHN for north-south, HHE for east-west). These data were then compared to the hydroseismograms from boreholes S13 and S14.

To visualize and inspect the waveforms, we used the SeisGram2K Viewer software (http://alomax.free.fr/seisgram/SeisGram2K.html). SeisGram2K is a Java-based program designed for interactive visualization of seismic recordings. It can be used either online or as a downloadable software, allowing for local analysis without an internet connection.

SeisGram2K can display one or more sets of seismograms, either as single traces or three-component records.

### Recognizing seismic events from HPD measures

Based on the above illustrated criteria, we excluded events that were unlikely to be detected by the aquifer. This involved associating a range of magnitudes with a corresponding range of distances from the horizontal boreholes S13 and S14.

From the global seismic catalog of the INGV, we selected 1068 events recorded by the GIGS station, located approximately 250 m from boreholes S13 and S14. This station, part of the GINGER experiment^48^, continuously monitors microseismicity in the Gran Sasso region and records global seismicity.

A visual screening of the selected events was conducted by comparing seismograms from the Gran Sasso National Laboratories (LNGS-INFN) with hydroseismograms from the continuous water pressure recordings of channels ch2 and ch3 in boreholes S13 and S14. Only events clearly identifiable in the hydroseismograms were considered, excluding those below the noise level.

In many cases, visual inspection alone was insufficient to identify coincidences. Therefore, spectrograms were used to complement the analysis. A Butterworth filter was applied to generate time-frequency spectrograms, mitigating the Gibbs effect and ensuring minimal edge distortion.

By visually examining color variations in the spectrograms, anomalies in the hydroseismograms were identified. These anomalies were then compared to the corresponding seismograms from the GIGS station, considering factors such as period, peak amplitude, peak-to-peak amplitude, wavelength, and temporal alignment of wave arrivals.

### Statistical analysis of sensitivity

The HPD sensitivity was conducted by comparing the number of events identified by the HPD with those recorded by the nearby seismic station GIGS (1068 events) for various magnitude and epicentral distance values. The analysis was performed both considering all recorded events and by separately investigating crustal and deep earthquakes. In our data, as depth values appear as two clusters separated by the depth value of ca. 35 km (Sect. 4.3.), the threshold distinguishing crustal from deep earthquakes was conventionally set at this latter value. We emphasize that the sensitivity considered here is that of the HPD relative to GIGS, meaning we are not estimating the probability that the HPD detects an earthquake given the occurrence of the event, but rather the probability that the HPD detects an earthquake given that it was detected by GIGS. It is excluded that there are events detected by the HPD that are not detected by the GIGS station, as this circumstance has never occurred in our data. Therefore, if we view the HPD data as a test, then the specificity (i.e., the ratio between the number of events not detected by the HPD and those not detected by GIGS, either because they are too weak or because they do not exist) is always equal to 1. Hence, we need to estimate only the sensitivity. This latter is estimated as the ratio between the number of events detected by the HPD and those detected by GIGS. We investigated the relationship between sensitivity, magnitude, and epicentral distance of earthquakes. Denoting by M the magnitude and by z the natural logarithm of the epicentral distance, the sensitivity was estimated, for crustal and deep earthquakes, at the nodes of a regular grid with a step of 0.5 in the (M, z) plane. For each node, the estimate was calculated considering the events within a unit diameter circle in the (M, z) plane (Sect. 4.3.). To represent the sensitivity threshold with a simple linear relationship in the (M, z) plane, we calculated the line that identifies points where the probability of success (detection of an earthquake by the HPD) is equal to a conventional value of 0.5.

## Results

### Comparison between seismic signals from GIGS station and hydroseismograms for some main seismic events

**Fig. 6:**
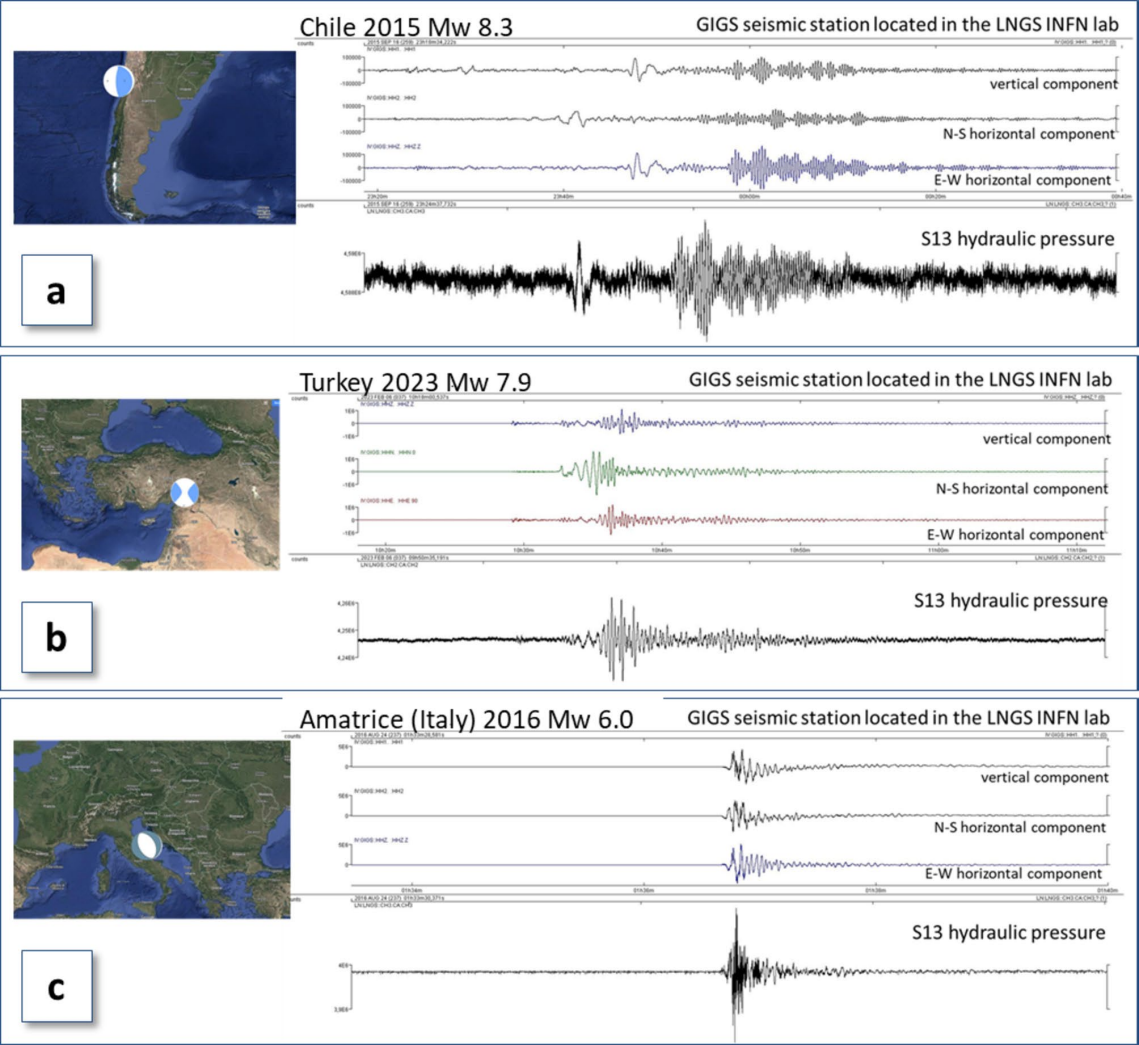
Hydroseismogram (S13 hydraulic pressure data) vs. seismogram (GIGS seismic station located in the LNGS nearby to S13 site): two teleseisms (**a** and **b**) and local event (**c**). The top traces are the vertical, N-S and E-W components of Trillium 240 s sensor (GIGS) and the bottom trace is the water pressure signal; **(a)**: Chile earthquake of September 16th, 2015 (offshore Coquimbo, Mw 8.3, epicentral distance: 11800 km); **(b)**: Turkey earthquake of February 6th, 2023 (Adana area, Mw 7.9, epicentral distance: 20 km); **(c)**: Amatrice earthquake (central Italy) of August 24th, 2016 (Mw 6.0, epicentral distance: 39 km). The plots of signals have been performed by means of the free software SeisGram2K (version 7.0) and the maps, using Google Earth Pro version 7.3.6. (https://www.google.it/earth/versions/).

A comparison of seismic traces recorded by our HPD with those obtained from the GIGS seismic station for high-magnitude seismic events highlights the potential of this instrument for seismic monitoring. Figure 6 compares seismic signals recorded for major events with varying epicentral distances and focal mechanisms: the September 16, 2015 Chile earthquake, the August 24, 2016 Amatrice (Central Italy) event, and the February 6, 2023 Turkey-Syria earthquake. Notably, as the epicentral distance increases, the duration of the seismic trace and the time scale significantly increase.

The Chile event, with a magnitude of Mw 8.3 and a hypocentral depth of approximately 22 km, was associated with a reverse fault mechanism and was located about 11,800 km from our HPD site. The Amatrice earthquake had a magnitude of Mw 6.0 and a hypocentral depth of about 8 km, was associated with a normal fault, and was located 39 km away. Finally, the Turkey-Syria event had a magnitude of Mw 7.9 and a hypocentral depth of approximately 20 km, was associated with a strike-slip fault mechanism, and was located about 2000 km from our HPD site. For these high-magnitude events, the signal recorded by the HPD is visibly congruent with those recorded by the GIGS seismic station, for both body waves and surface waves (Fig. 6).

### A relevant example of the conducted analysis

To illustrate the analysis process, consider the earthquake that occurred in Mongolia on January 11, 2021, at 21:33:01 UTC, with a magnitude of 6.7 and an epicentral distance of approximately 6,314 km from the boreholes.

The first step involved visually inspecting the hydroseismogram for the time interval corresponding to the potential event. In this case, no apparent anomalies were immediately visible in the 18:00–24:00 time window.

As mentioned previously, it is essential to account for time differences between the seismic station and the pressure sensor. Even after considering this time lag, no clear correlation with the seismic event was found.

**Fig. 7:**
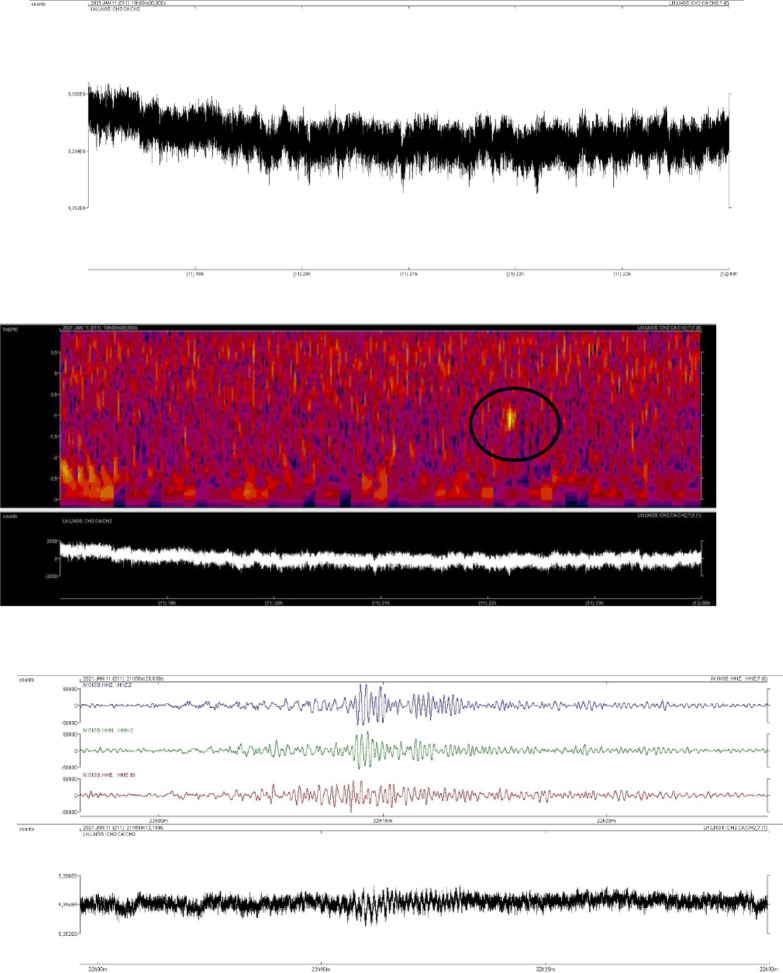
Example of data processing.

To facilitate the identification of potential anomalies, a spectrogram was generated. This particular case was chosen as an example because the anomaly was easily identifiable (Fig. 7) and appeared as a single, distinct feature. In contrast, many other cases involved multiple anomalies, making the analysis more complex.

Once the chromatic anomaly was located in the spectrogram, the corresponding time interval in the hydroseismogram was examined. The signal from the potential event in the hydroseismogram was then compared to the three components (vertical, north-south, and east-west) recorded by the GIGS seismic station. The time delay, as well as the traces from the seismic station, were consistent with the water pressure signal (Fig. 7). This confirmed the detection of the event by the aquifer, allowing us to measure and record the desired parameters, including signal duration, maximum and minimum counts, peak-to-peak amplitude, event duration in seconds, and conversion to bar.

### Hydraulic pressure versus seismic data

Between May 2015 and December 2023, our hydraulic pressure device (HPD) successfully identified 148 out of 1068 analyzed earthquakes. To be classified as a detected event, a clear hydraulic pressure signal corresponding to a hydroseismogram must coincide temporally with a seismogram recorded by the GIGS station (Figs. 6 and 7).

**Fig. 8:**
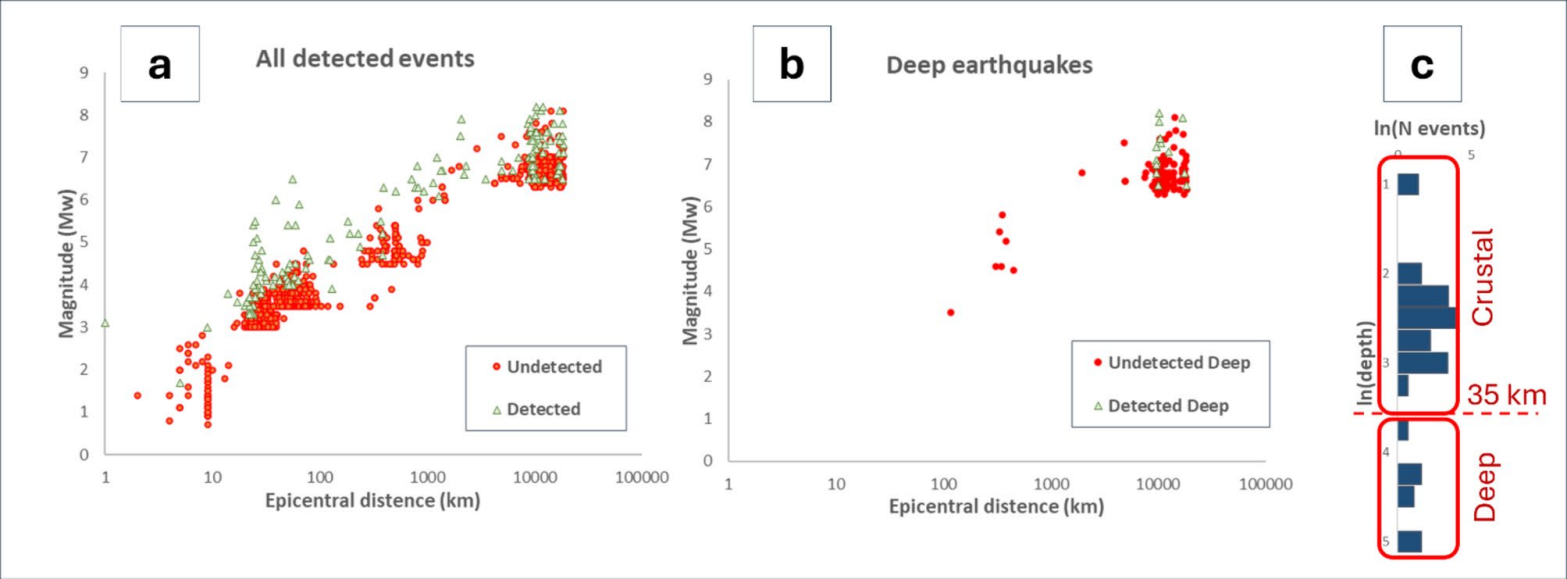
(**a**) Scatterplot of magnitude vs. distance for all detected earthquakes. (**b**) Scatterplot for deep events (depth > 35 km). Green and red dots denote detected and undetected events by the HPD, respectively (**c**) Histogram of depth values. Two main clusters can be recognized (crustal and deep events), separated by the depth value of 35 km.

**Fig. 9:**
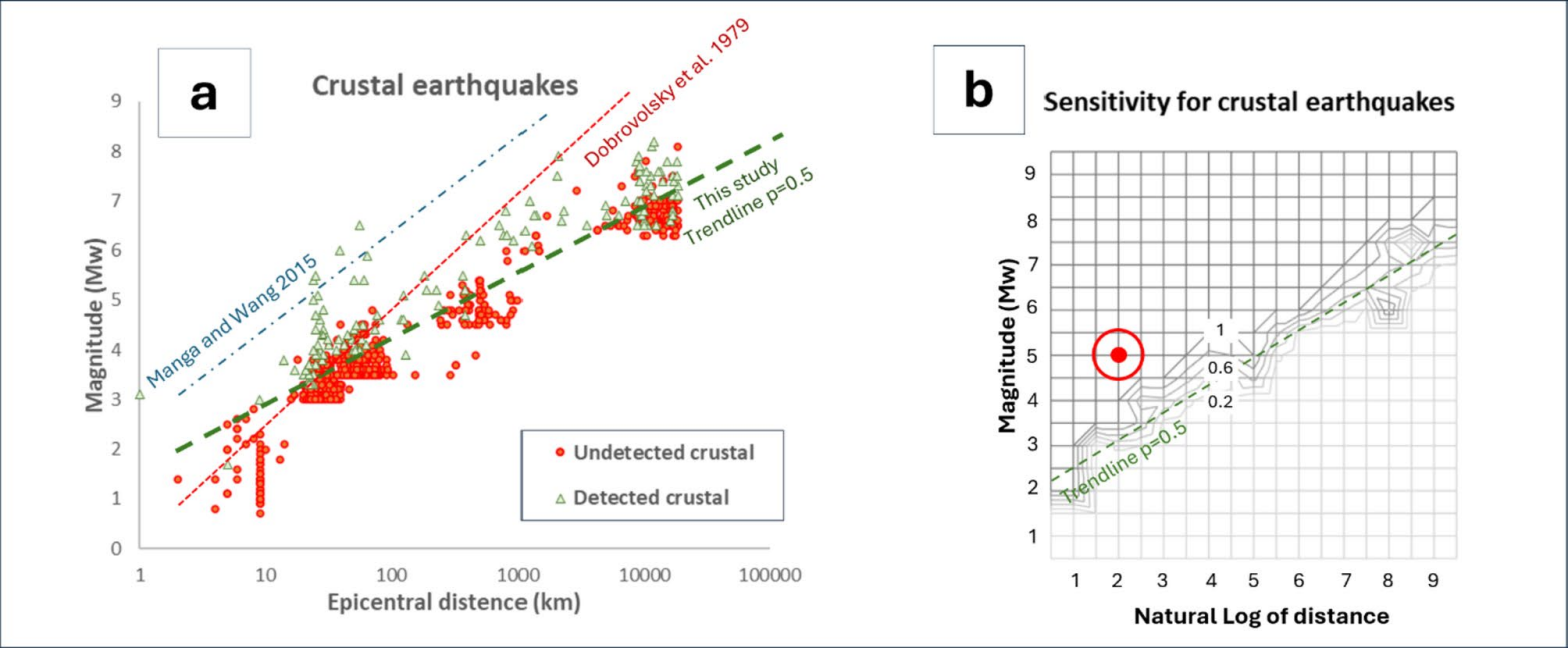
(**a**) Scatterplot for crustal earthquakes; blue dashed line denotes the detection limit from^13^ and red line represents the theoretical limit derived by Montgomery and Manga^12^ according to the Dobrovolsky et al.^50^ criterion. The green dashed line denotes the sensitivity p of 50% individuated in the diagram of panel (b). This threshold has been used here to conventionally identify the detection limit for our HPD. (**b**) Sensitivity map of HPD. Each isoline denotes points of the plot showing the same probability p of detecting by HPD events detected by the GIGS. By way of example, the node with coordinates (2, 5) is highlighted by a red dot, and the related circle with unit diameter, within which the events are counted, by a red circle. The dashed line is individuated as trendline of the isoline *p* = 0.5.

To comprehensively analyze these findings, we plotted the relationship between earthquake parameters and HPD detection for all detected events (Fig. 8a), and separately for deep (Fig. 8b) and crustal (Fig. 9a) earthquakes. Figure 9b shows the contour map of sensitivity values for crustal earthquakes, based on the ratios between number of events detected by the HPD and those detected by GIGS, calculated around each node of the mesh grid in the diagram. By way of example, at the node with coordinates (2, 5) highlighted by a red dot, the sensitivity is estimated as ratio of the number of events detected by HPD against those detected by GIGS, for all events falling within the red circle, with unit diameter. Then, once calculated the contour lines, the trendline of that related to probability *p* = 0.5 (dashed line in Fig. 9a and b) has been conventionally indicated as detection limit for HPD.

The previous state-of-the-art is represented by the blu dashed line, which defines a detection limit for seismic events based on earlier studies^13^ and the red dotted line, which denotes the more interesting theoretical limit individuated by Montgomery and Manga^12^ according to the Dobrovolsky et al.^50^ criterion. This latter can be viewed as a strong detection limit for a hydroseismic signal (Sect. 5).

## Discussion

Statistical analysis revealed that the HPD employed in this study exhibits significantly greater sensitivity compared to previous analogous studies^12,13,50^ and references therein). Overall, the HPD demonstrated a 13.9% success rate, detecting 148 seismic events out of 1068 identified by GIGS. However, this probability is strongly dependent on the magnitude and epicentral distance of each event, as illustrated in Figs. 8 and 9. A more detailed analysis conducted separately for crustal and deep earthquakes allowed us to identify a sensitivity threshold in the (M, z) plane, where M and z denote the magnitude and natural logarithm of the epicentral distance, respectively, of the events recorded by GIGS (Fig. 9b; Table 1). Above this threshold, success probabilities rapidly increase towards 100%. For crustal earthquakes, the HPD recorded 134 events out of 937, with an average success probability of 14.3% (95% Confidence Interval (CI): [12.2%, 16.7%]). The sensitivity of the HPD relative to GIGS for shallow events, estimated as the ratio of the number of events detected by the HPD to the number of events detected by GIGS (Sect. 3.2), is plotted as a function of M and z in Fig. 9b. Each isoline in this graph identifies the set of points in the (M, z) plane for which the same success probability is estimated. The trendline, indicated by a dashed green line, was identified as the least squares line of the isoline associated with a value of 0.5 (i.e., 50% success probability). Note that below this threshold line, success probabilities decrease drastically, while above it they increase rapidly. Regarding deep earthquakes, the picture is more uncertain due to the scarcity of events with an epicentral distance less than 2000 km. Here, we set this distance as the threshold beyond which we conventionally define an event as distant. The HPD recorded 14 deep events out of a total of 131, showing a success probability of 10.7%. However, the earthquakes detected by the HPD are only distant ones. The HPD did not detect any nearby deep events, while GIGS recorded 12. For distant deep events, the HPD detected 14 out of 119, showing a success probability of 11.8% (95% CI: [7.2%, 18.9%]). This latter value is compatible with that for nearby deep events, as the probability of detecting n events out of 12, given a success probability per event of 11.8%, is a binomial variable. When *n* = 0, this probability is equal to 22.3%. Therefore, the circumstance of identifying zero events out of 12 would not be rare or unlikely. This does not allow us to state that the success probability is the same for nearby and distant events, but we simply cannot exclude it, since the sample of nearby deep events is too small to make a statement. Overall, the sample of deep events appears rather heterogeneous and clustered, however, Figs. 8 and 9 highlight that the sensitivity of the HPD for these events appears compatible with that observed for crustal earthquakes.

**Table 1:** Statistics comparing the number Of events detected by HPD and by GIGS for several earthquake categories. The probability Of success, i.e. Of detecting an event by HPD once it has been detected by GIGS, is denoted by a real number in the range 0–1, estimated as ratio between number Of events detected by HPD (N_HPD_) and by the GIGS (N_GIGS_). The 95% confidence intervals (CI) are estimated according to a binomial probability model.

	*N*. detected by HPD (NHPD)	*N*. detected by GIGS (NGIGS)	Success probability *p* = NHPD/NGIGS	Lower limit 95%CI	Upper limit 95%CI
All events	148	1068	0.139	0.119	0.161
Crustal	134	937	0.143	0.122	0.167
Deep	14	131	0.107	0.066	0.173
Deep far	14	119	0.118	0.072	0.189
Deep near	0	12	0	0	0.264

In summary, the data in Table 1 point out that the various event groupings (all events, crustal, deep, etc.) show similar values ​​of success probability, with all confidence intervals exhibiting a large degree of overlap. Only the set of deep and nearby seismic events shows an excessively wide confidence interval (which includes all the other estimated intervals) since the related data set is particularly poor.

Figure 9a compares our results to those of previous studies. The threshold identified in this study is 1–2 magnitude units lower than that determined by Manga and Wang^13^. Particularly noteworthy is the comparison with the threshold defined by Montgomery and Manga^12^. These authors pointed out that in a typical aquifer a volumetric strain of the order of 10^− 8^ is associated with a centimetric piezometric head change, which is barely detectable by high precision measurements. Then, they calculated the combination of magnitude and epicentral distance values associated with such a volumetric strain, following the criterion by Dobrovolski et al.^50^, interpreting them as a sensitivity threshold (red dotted line in Fig. 9a). This threshold can be viewed as a “hard” limit for detecting seismic events, as Montgomery and Manga^12^ in a large dataset analyzing the response of 912 wells to more than 44 seismic events, did not identify any events below this threshold.

Figure 9a shows that, for distant events, the HPD used in this study is significantly more sensitive than predicted by the Dobrovolski et al.^50^ criterion. This finding warrants further investigation, as it highlights aspects of the mechanisms underlying hydroseismic detection that are not yet fully understood and will be the subject of future studies.

Future research should focus on improving the HPD’s ability to detect deeper earthquakes while maintaining its sensitivity to shallow events. This could involve exploring different pressure sensor configurations, optimizing data processing techniques, or developing advanced signal processing algorithms. Additionally, a more comprehensive understanding of the relationship between seismic wave propagation and hydraulic pressure variations is essential for enhancing the HPD’s overall performance.

## Conclusions

Our investigation into the earthquake detection capabilities of HPD in wellbores in carbonate rock (boreholes S13 and S14), spanning from May 2015 to December 2023, yielded promising results in comparing the detection capability of the HPD to the currently published bibliography. The device accurately identified 148 out of 1068 analyzed seismic events. It is recalled that in this study we estimated the sensitivity of the HPD in comparison with that of the GIGS system, in other words, we estimated the conditional probability that a seismic event is detected by the HPD, given that it has already been detected by the GIGS. The sensitivity analysis involving crustal earthquake magnitude and epicentral distance has pointed out that our HPD significantly overcomes the sensitivity limit for seismic event detection reported by previous studies, for great epicentral distance^12,13,50^. It appears more effective also in detecting deep events, although our analysis was limited to far events, due to lack of deep earthquakes located near to the study area. It would be desirable to have a larger data set of nearby deep events in the future, to achieve a statistically significant estimate of the line identifying the sensitivity limit for deep earthquakes.

The Gran Sasso Aquifer (GSA), characterized by its complex hydrogeological and seismotectonic conditions, serves as an ideal environment to deploy the HPD, both in terms of middle-long term and high frequency pore pressure monitoring. Its strategic placement within the aquifer’s core, with two wells intercepting the main fault network, offers a unique opportunity to study the intricate interplay between hydrological processes and seismic activity.

Given the encouraging outcomes of this research, we are committed to ongoing monitoring efforts using the HPD to further explore its potential as a valuable tool for earthquake-related studies.

## Electronic supplementary material

Below is the link to the electronic supplementary material.

Supplementary Material 1

## Data Availability

Part of the data analyzed here is provided within the supplementary materials. The full data set can be provided by request to the corresponding author. e-mail: vincenzo.guerriero@univaq.it.
