# Supplementary material for: Hydroseismograms at Gran Sasso aquifer, central Italy, for earthquake hydrology studies
Source: Sci Rep. 2025 Apr 16;15:13162. doi: 10.1038/s41598-025-96113-4 (PMC12003777; doi:10.1038/s41598-025-96113-4)

## Slide 1
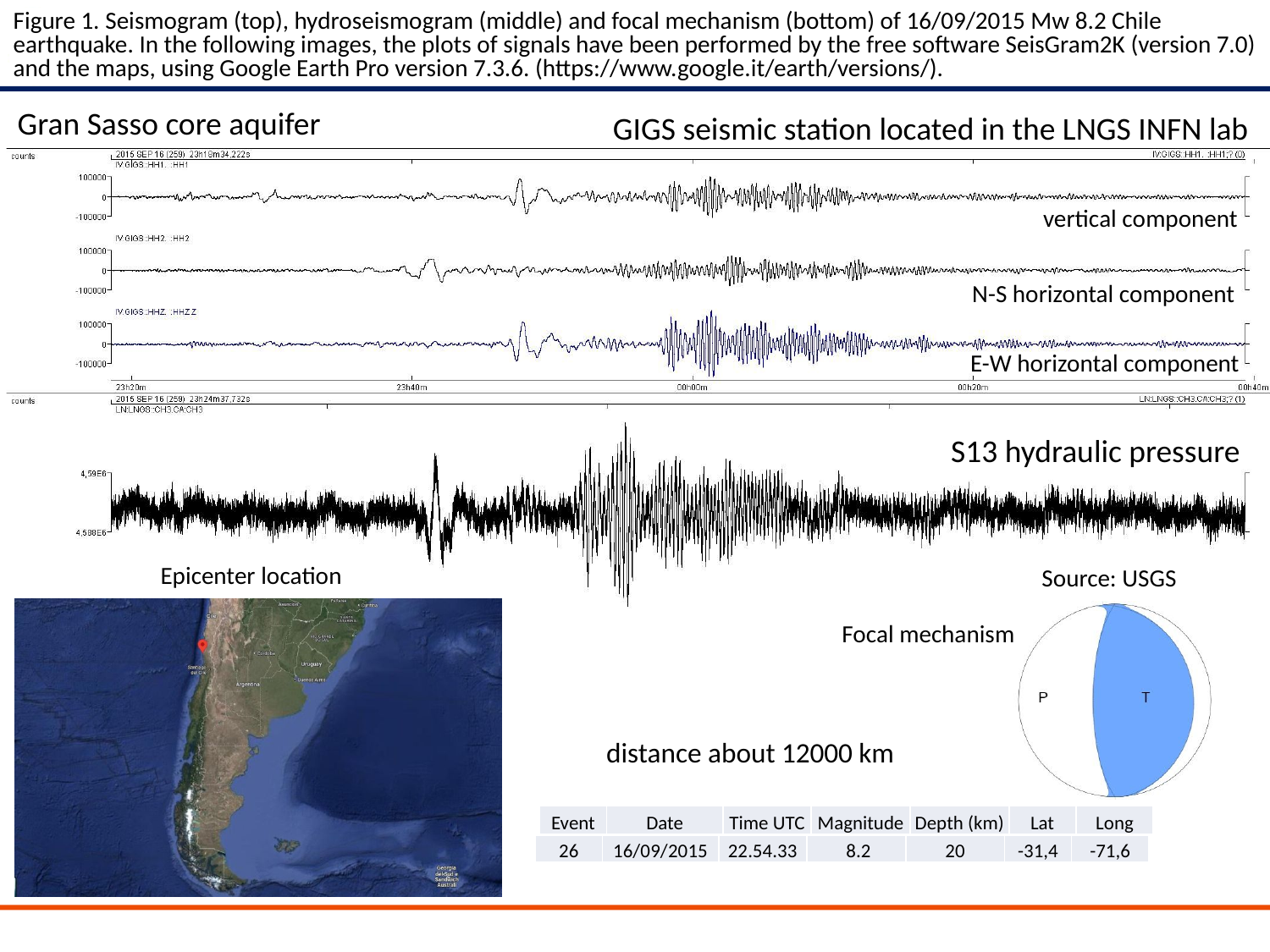

## Slide 2
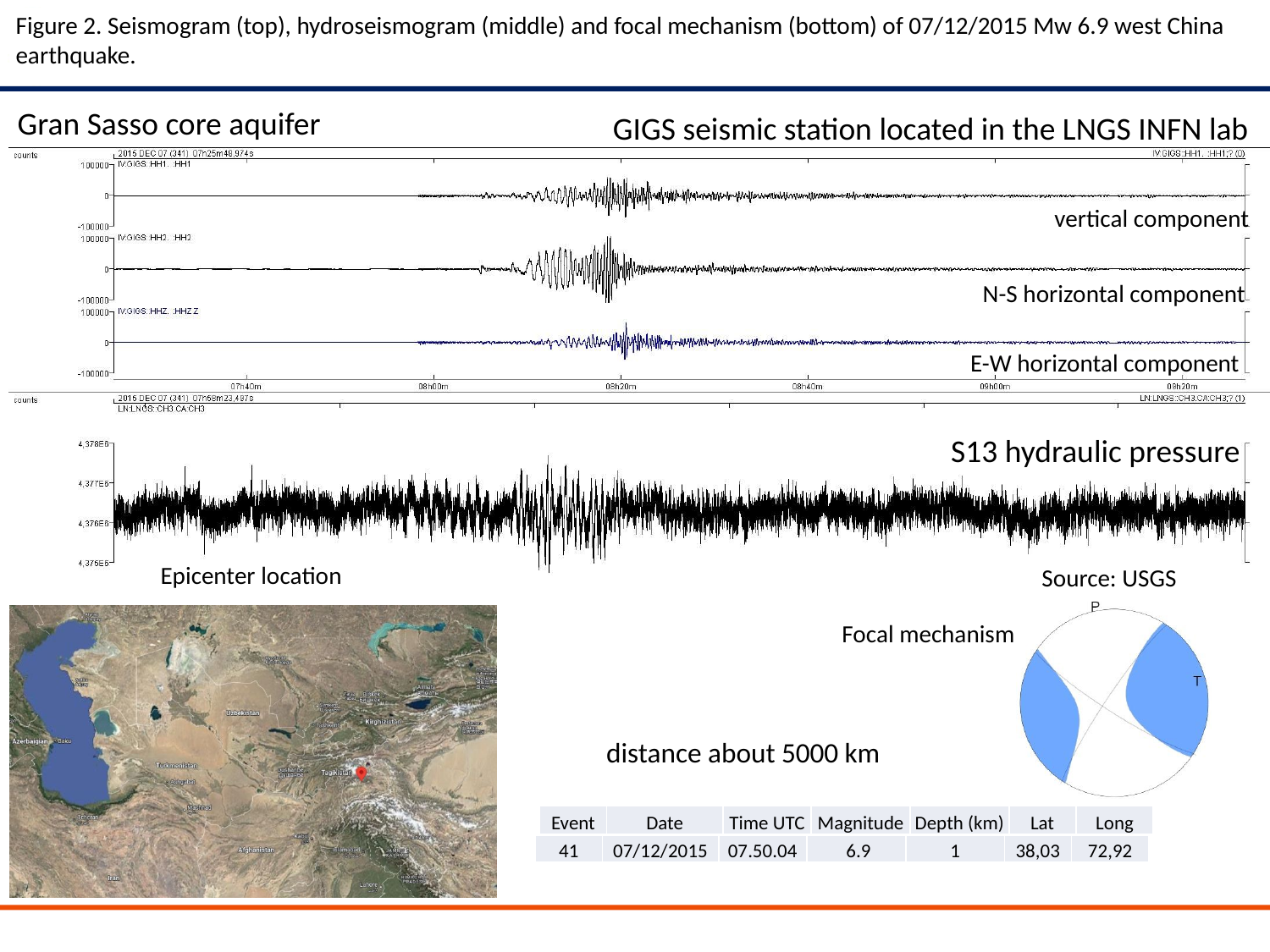

## Slide 3
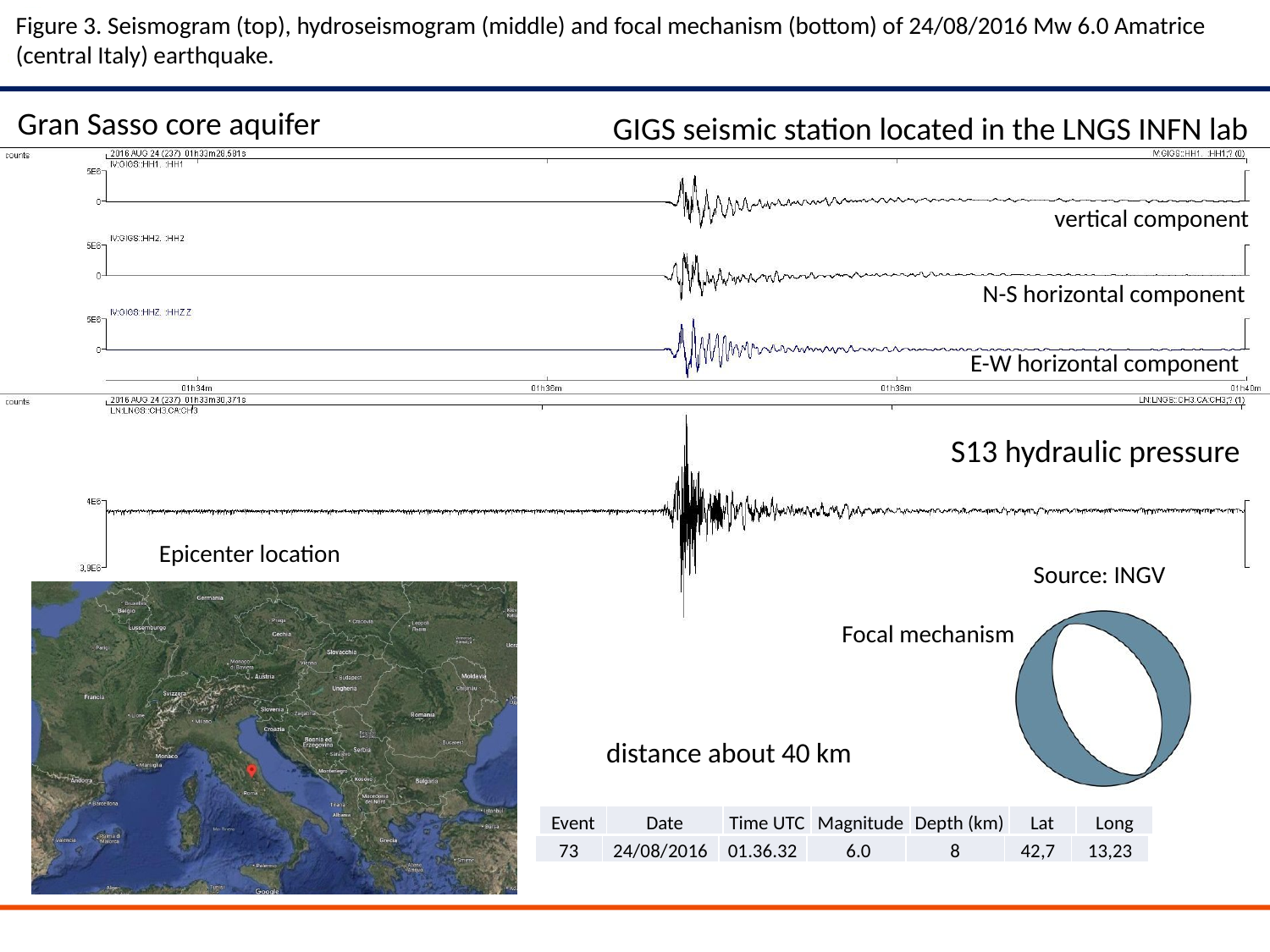

## Slide 4
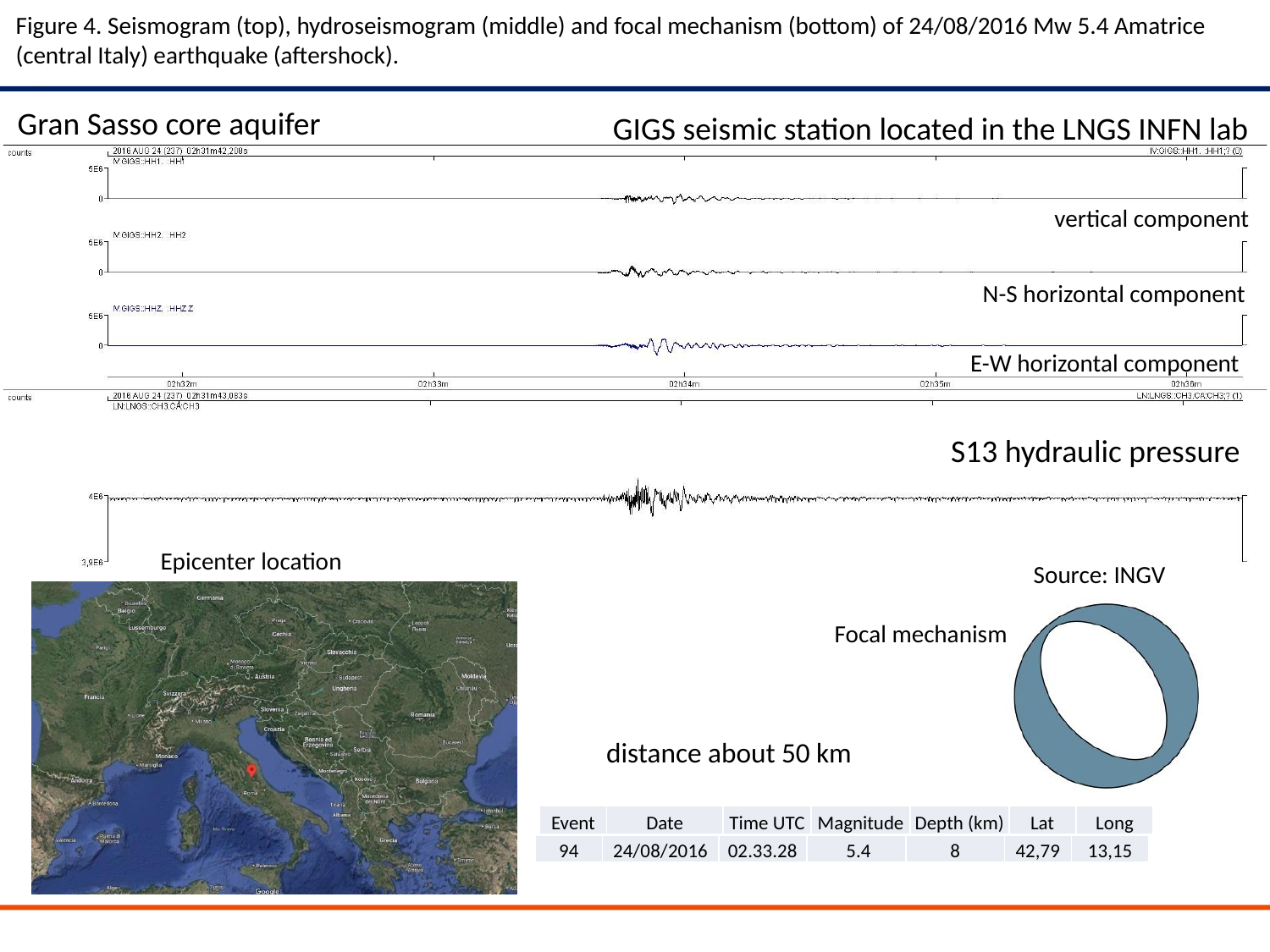

## Slide 5
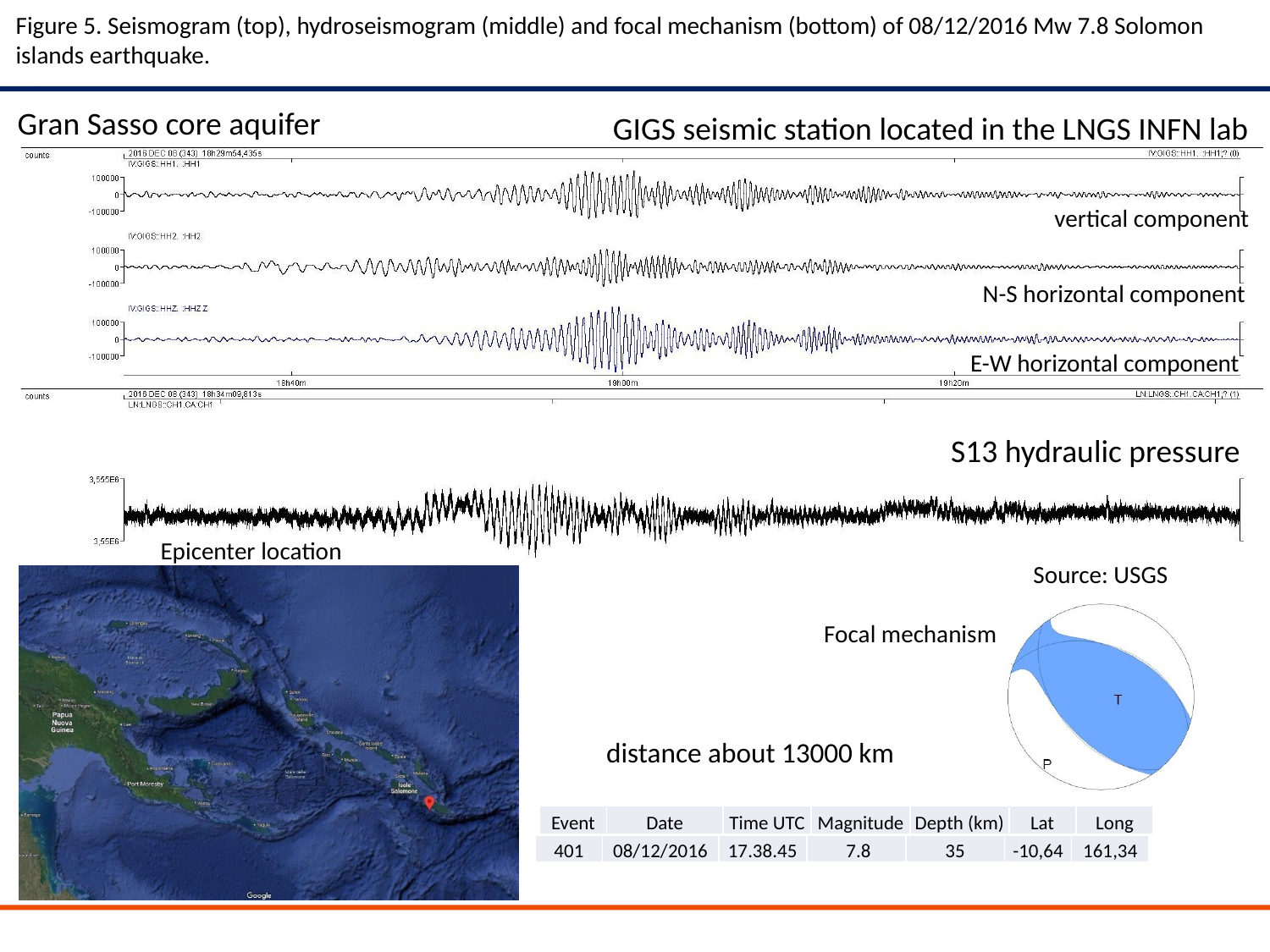

## Slide 6
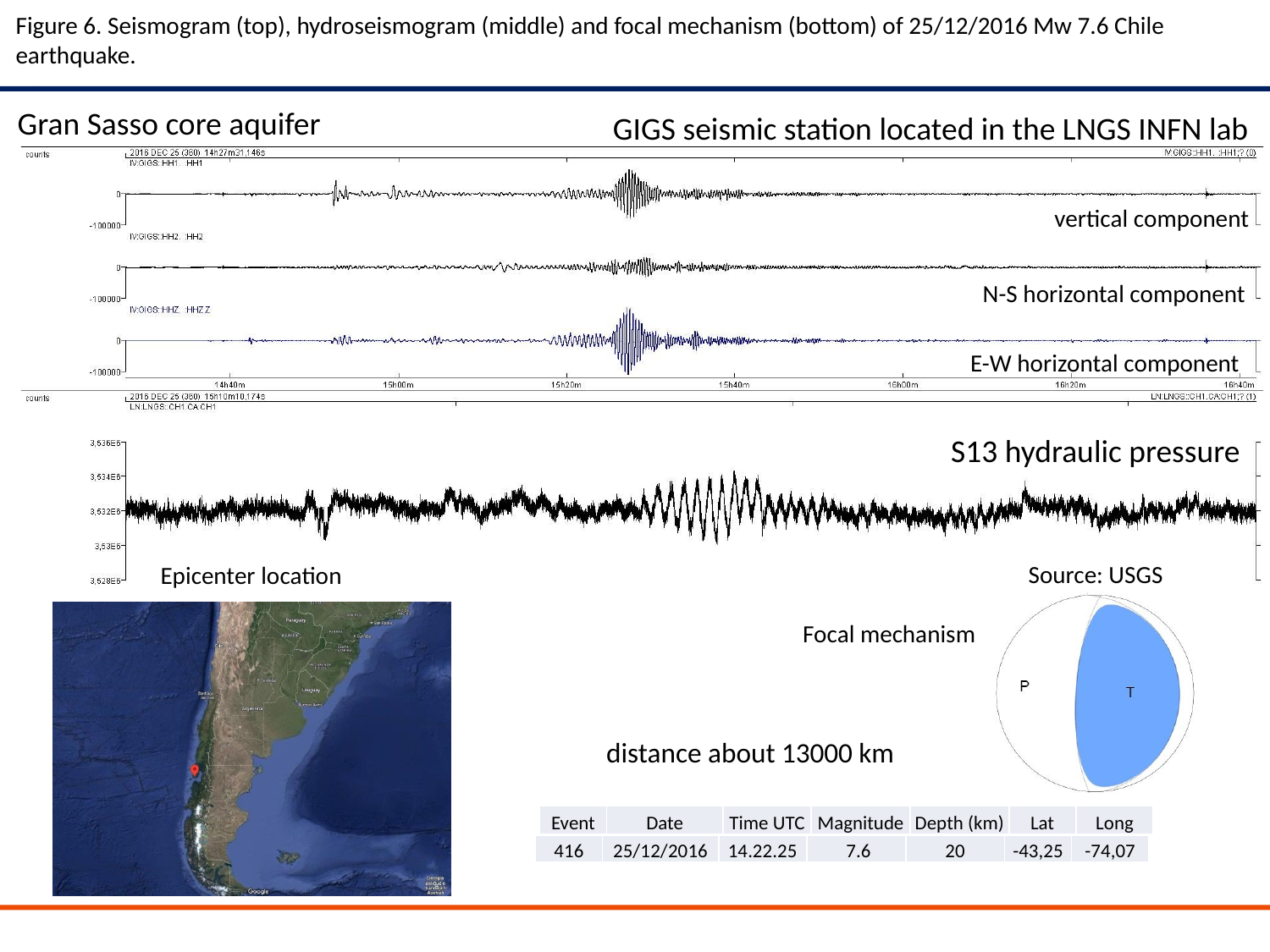

## Slide 7
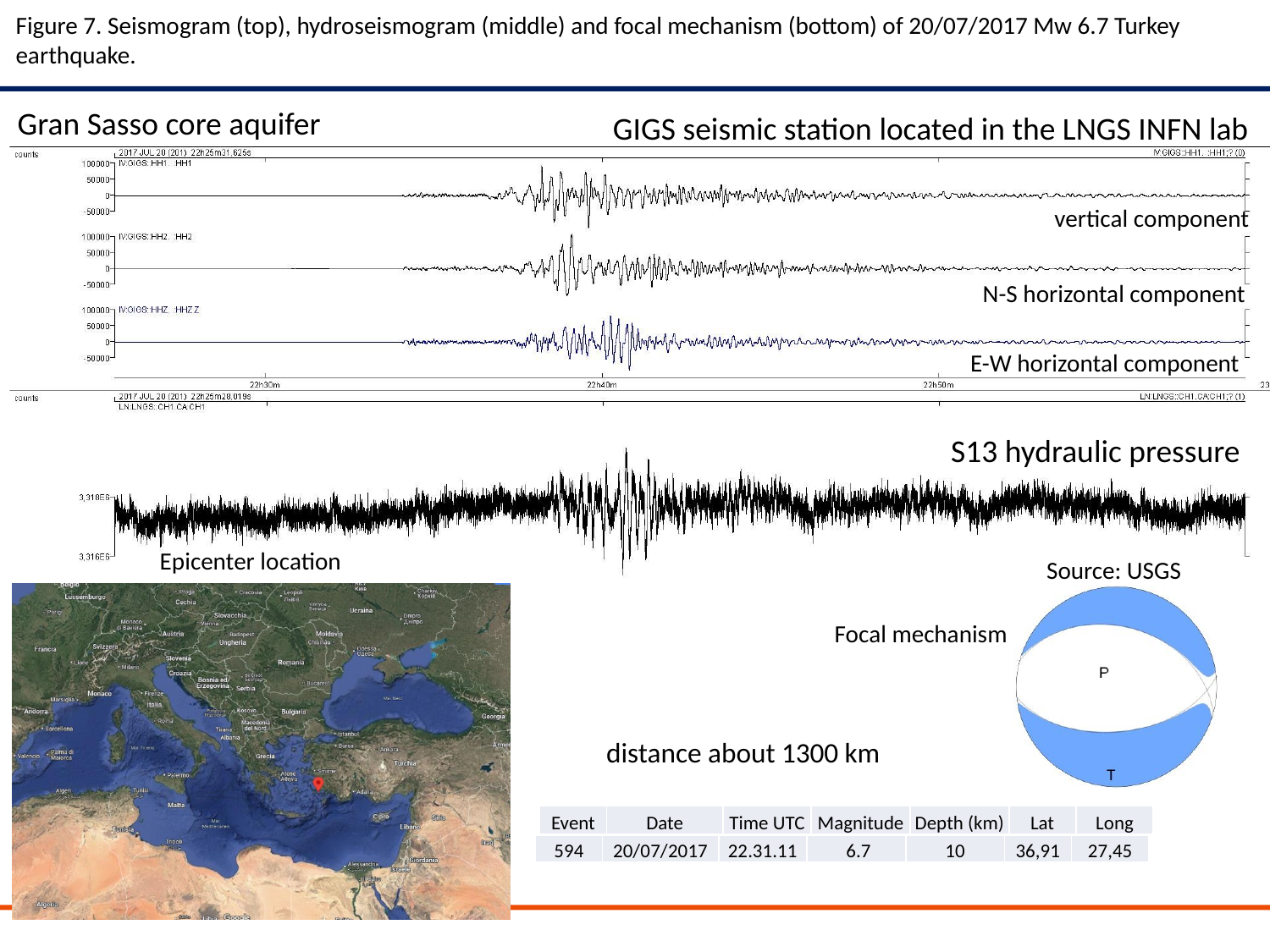

## Slide 8
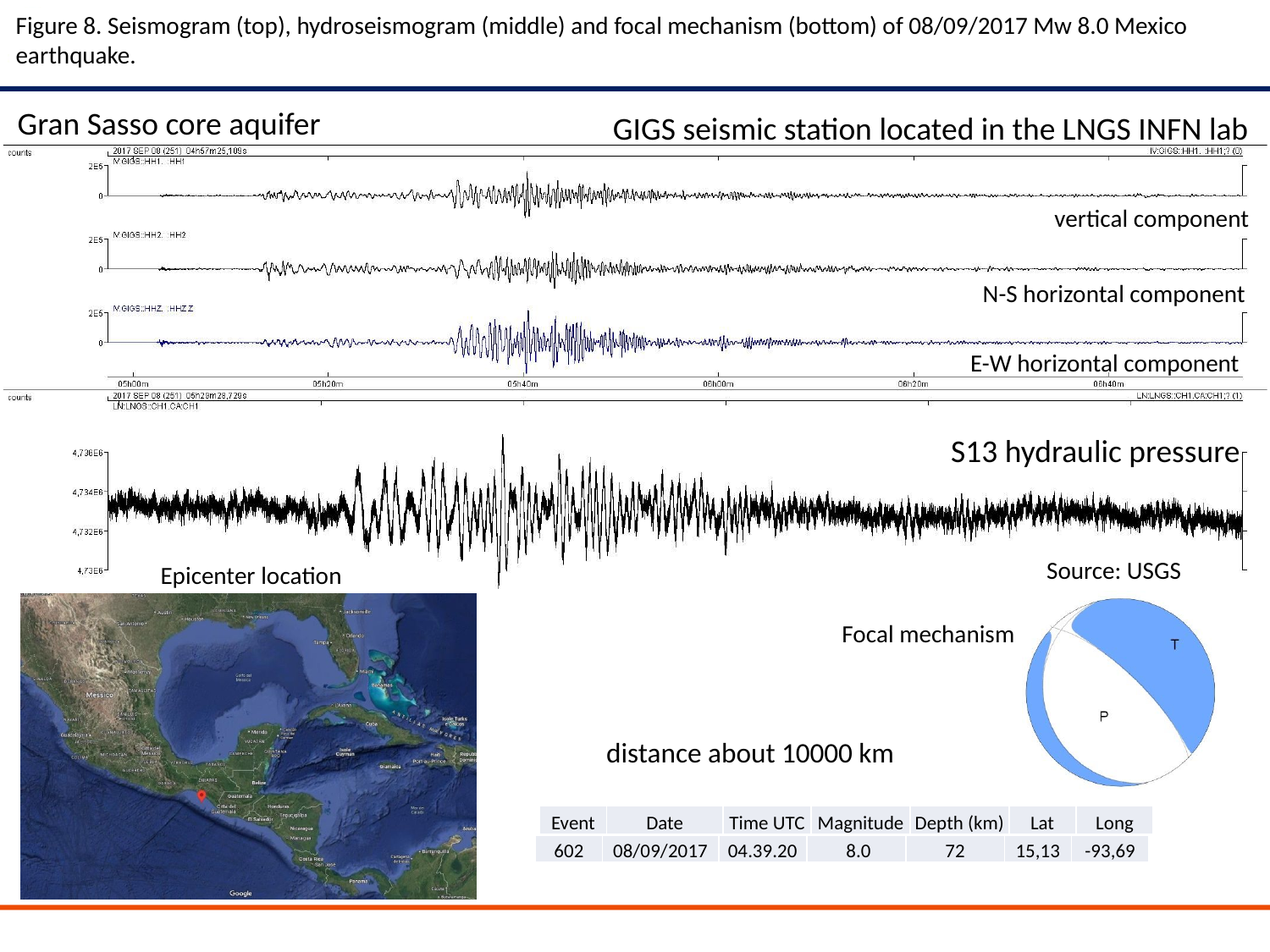

## Slide 9
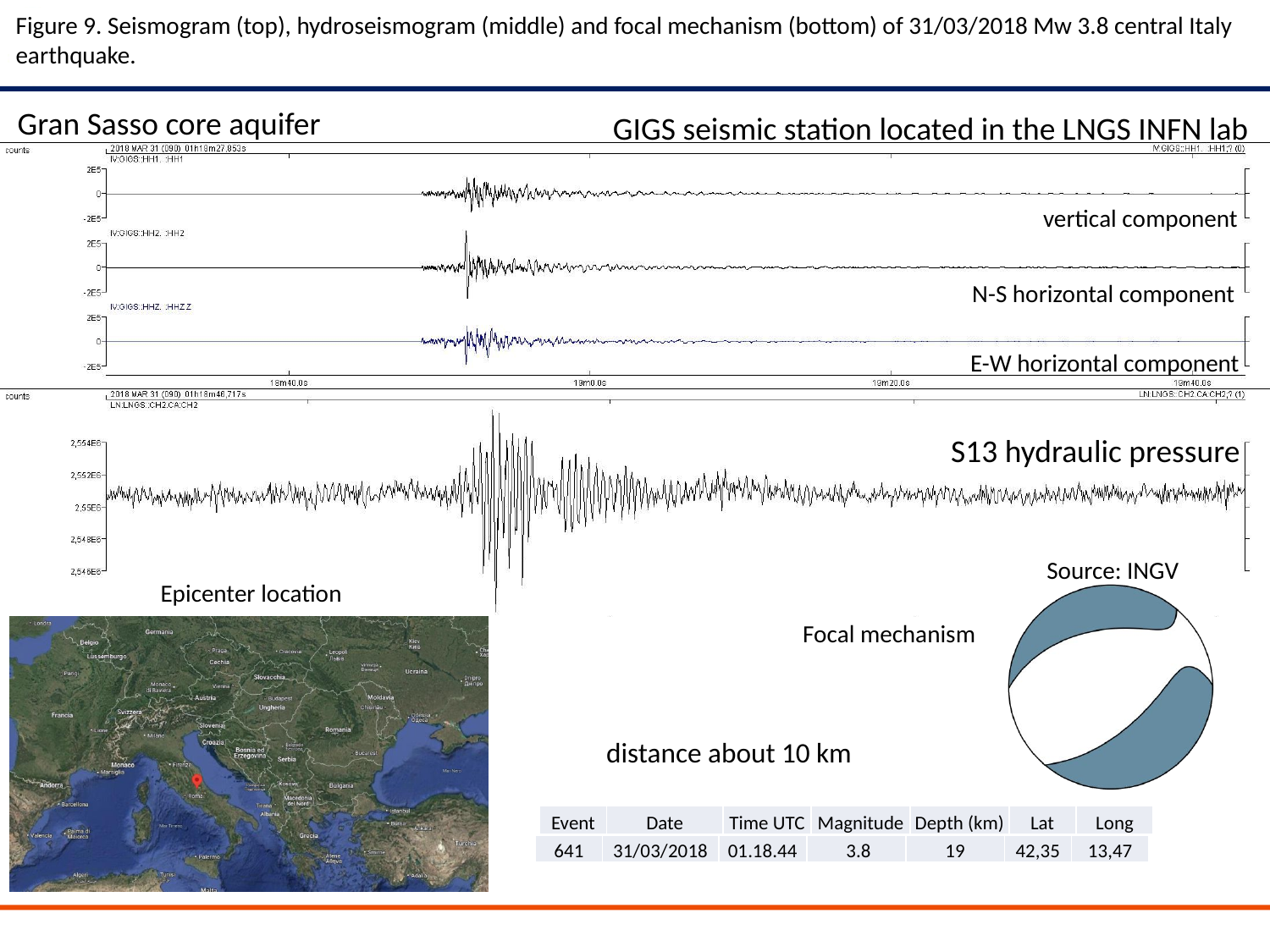

## Slide 10
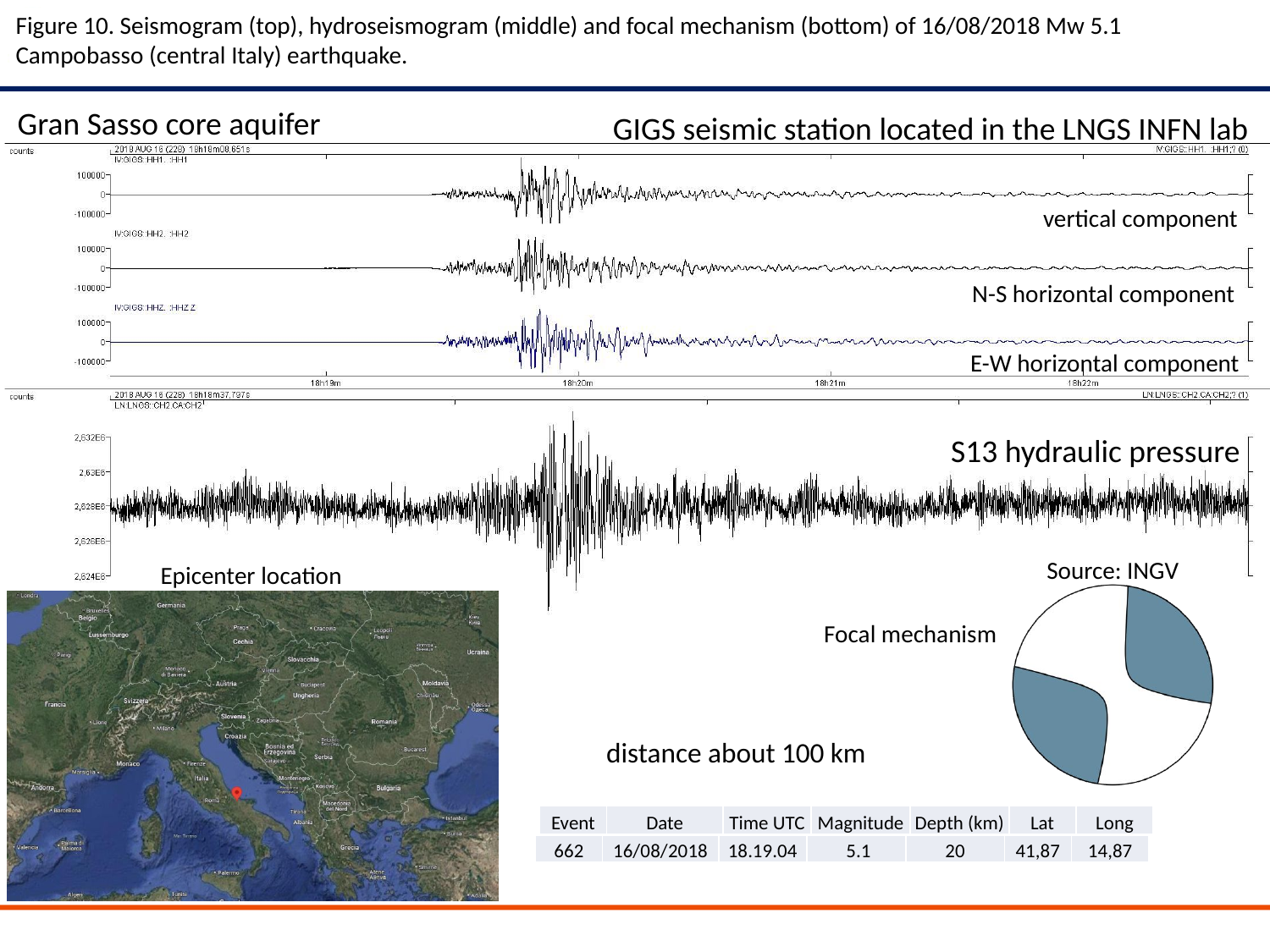

## Slide 11
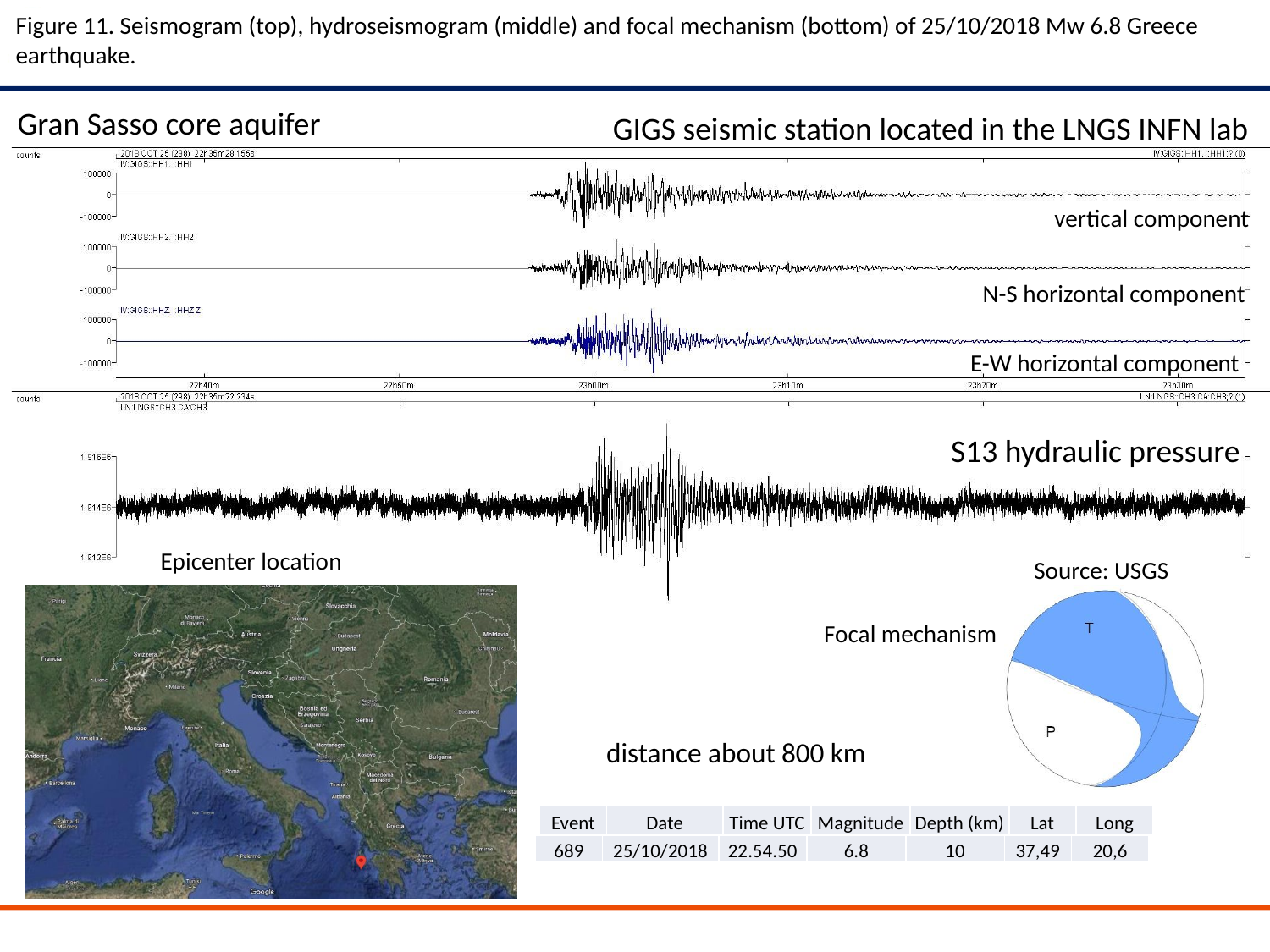

## Slide 12
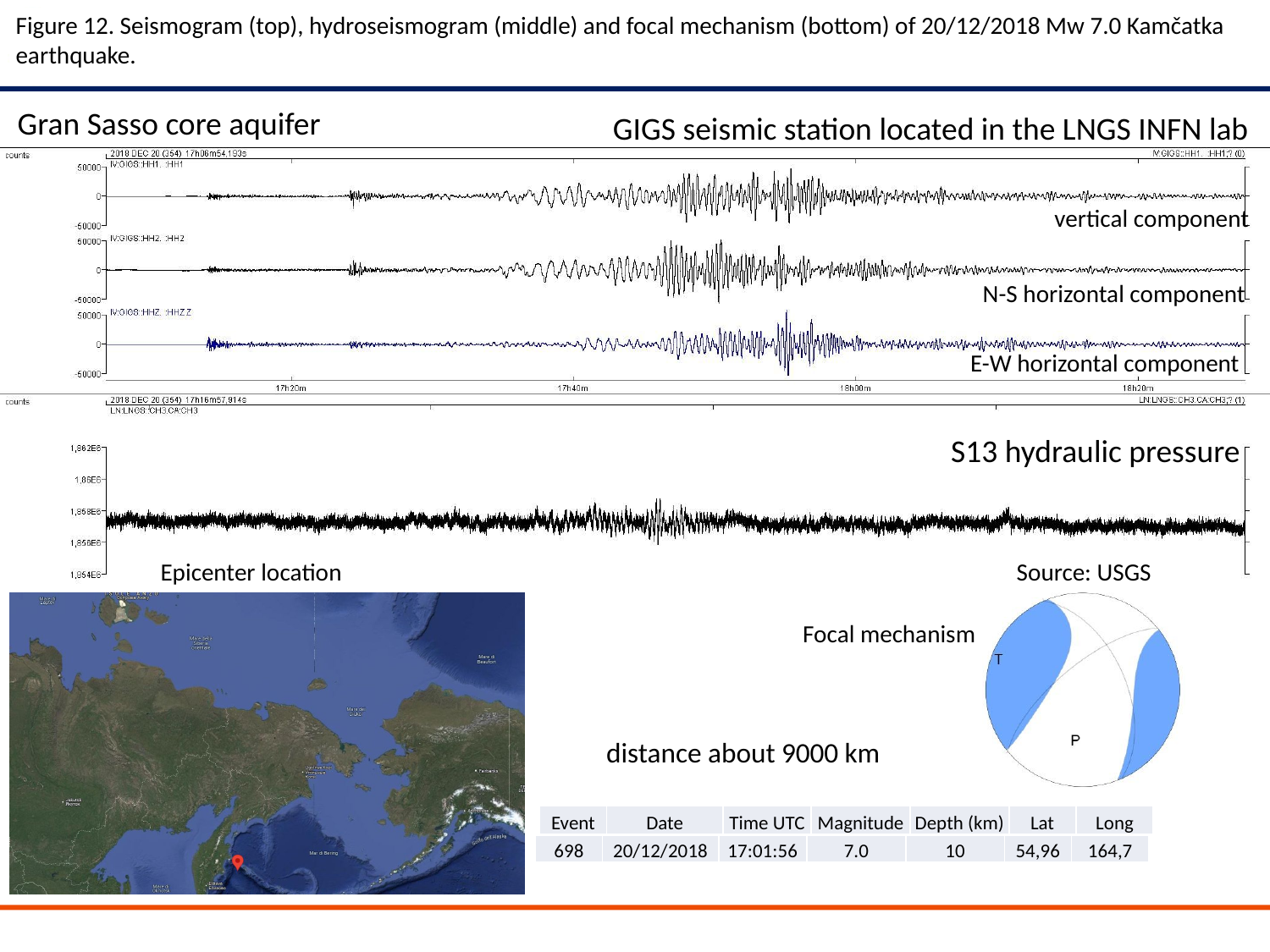

## Slide 13
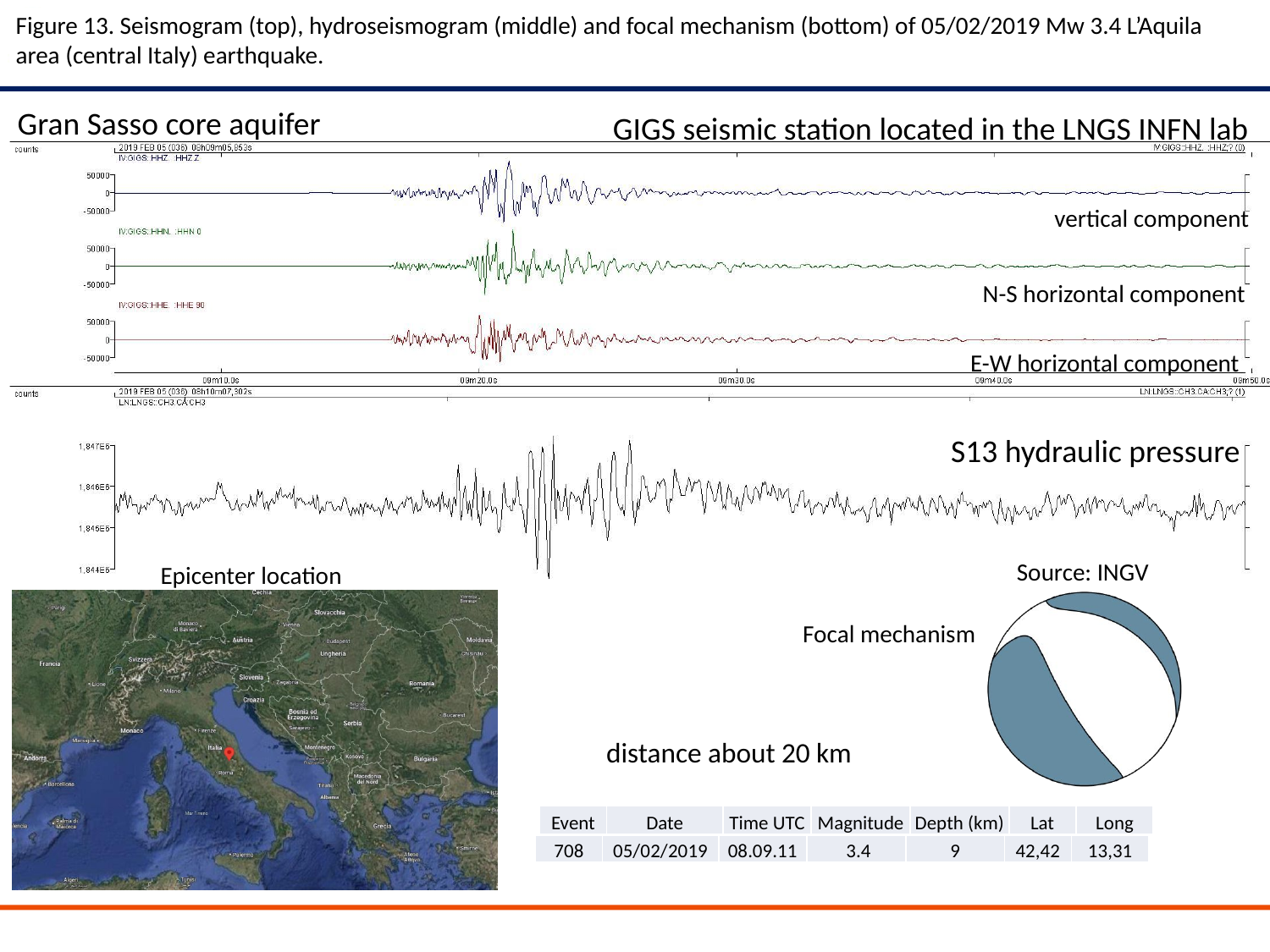

## Slide 14
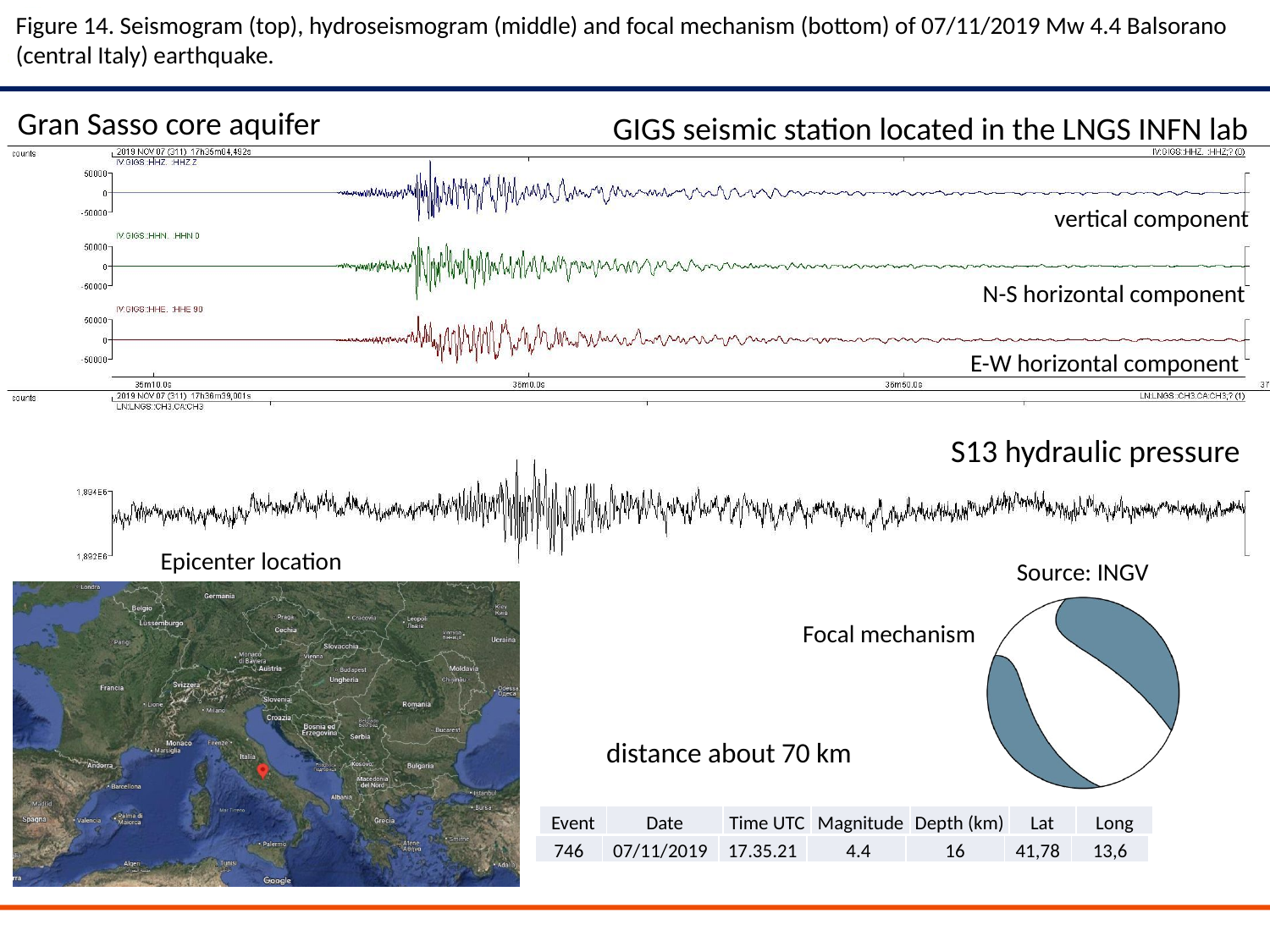

## Slide 15
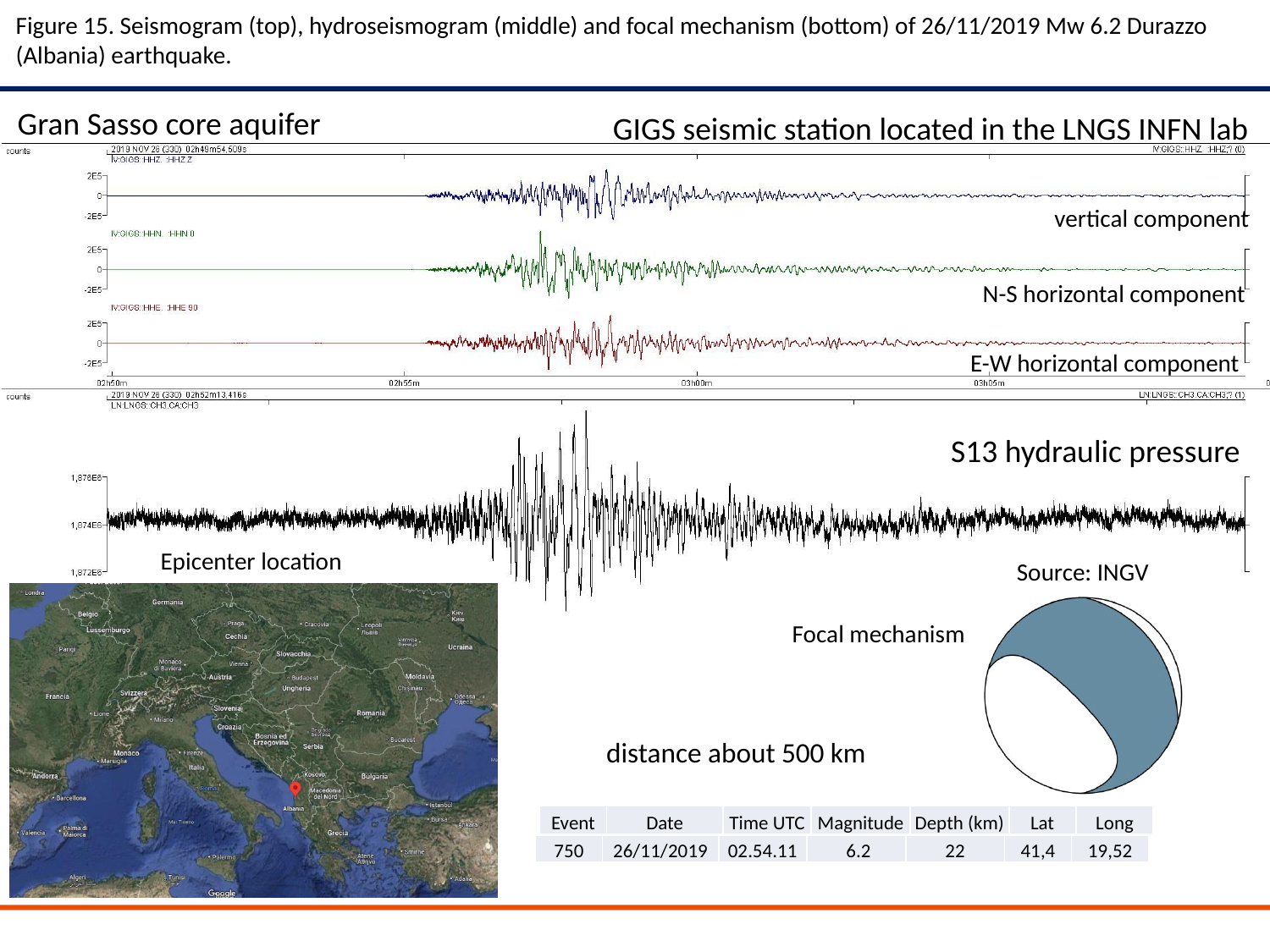

## Slide 16
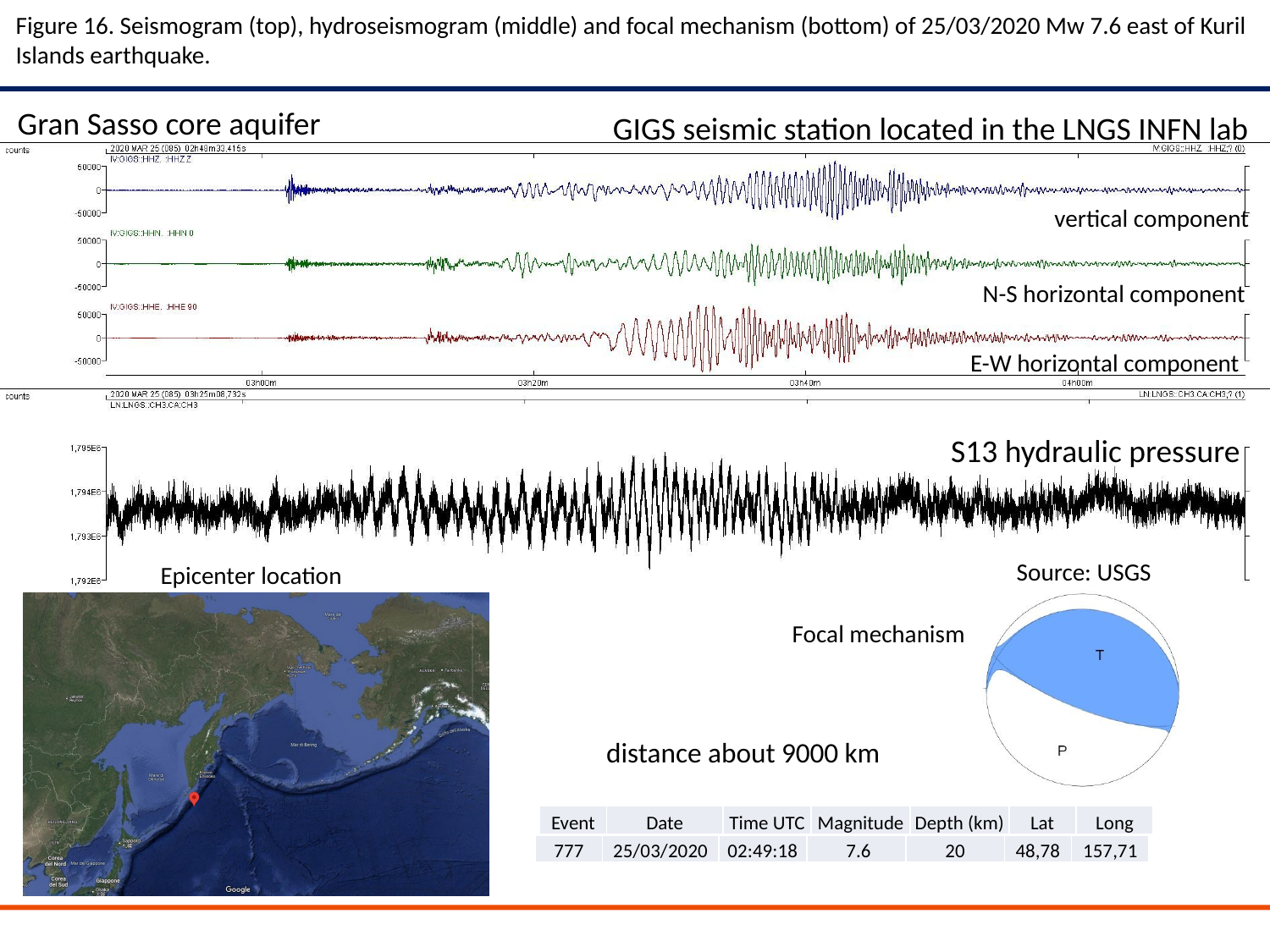

## Slide 17
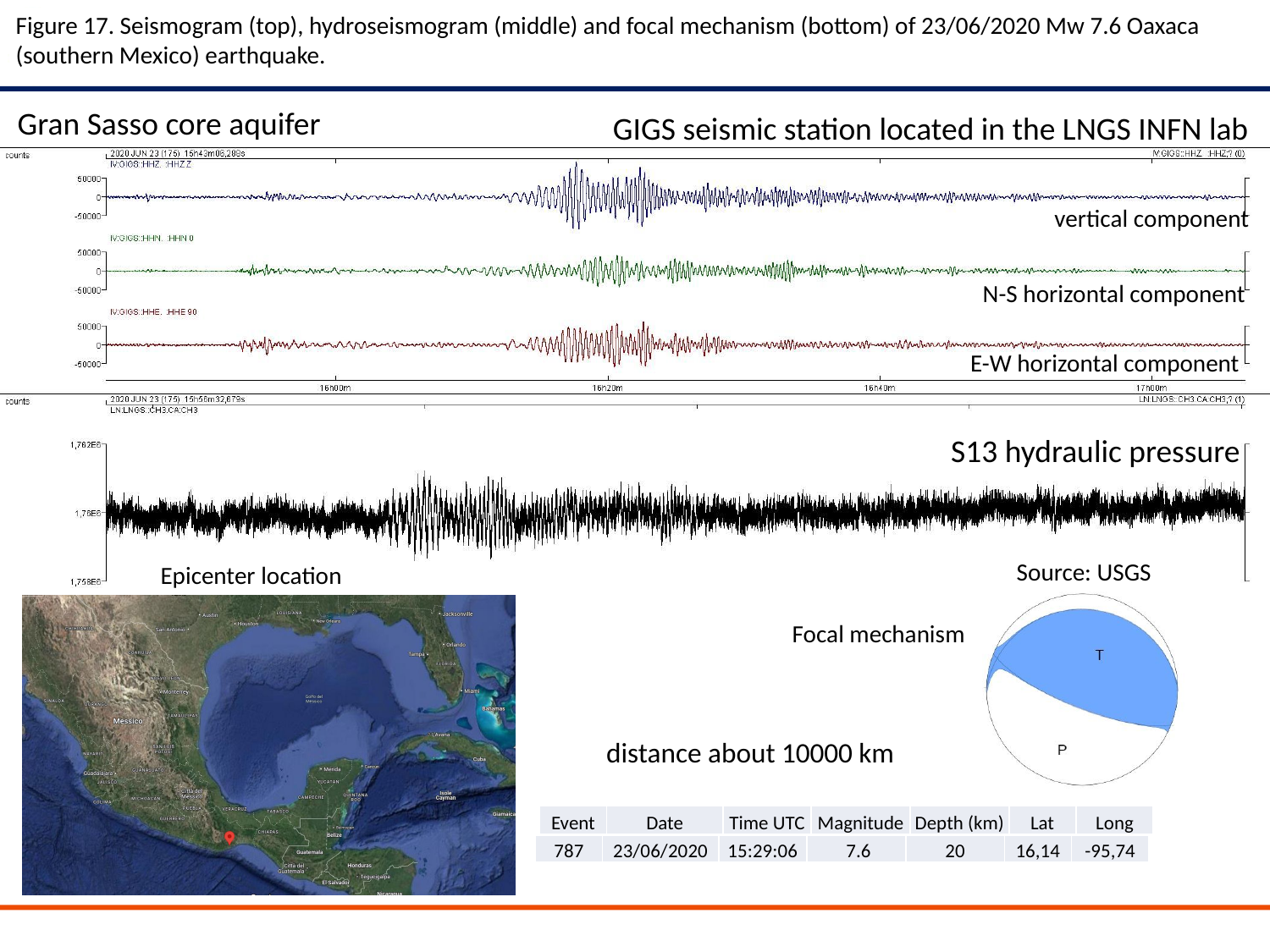

## Slide 18
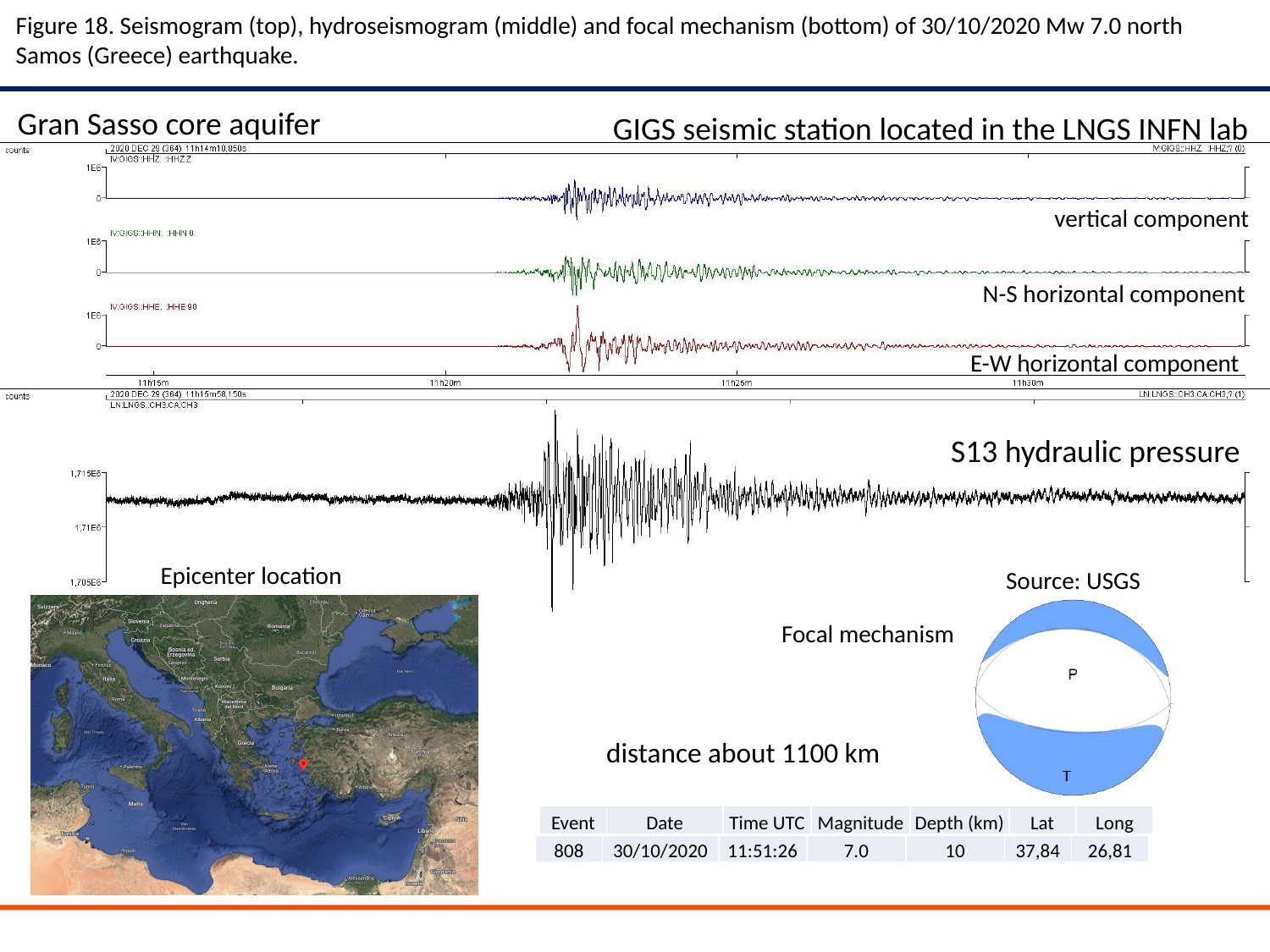

## Slide 19
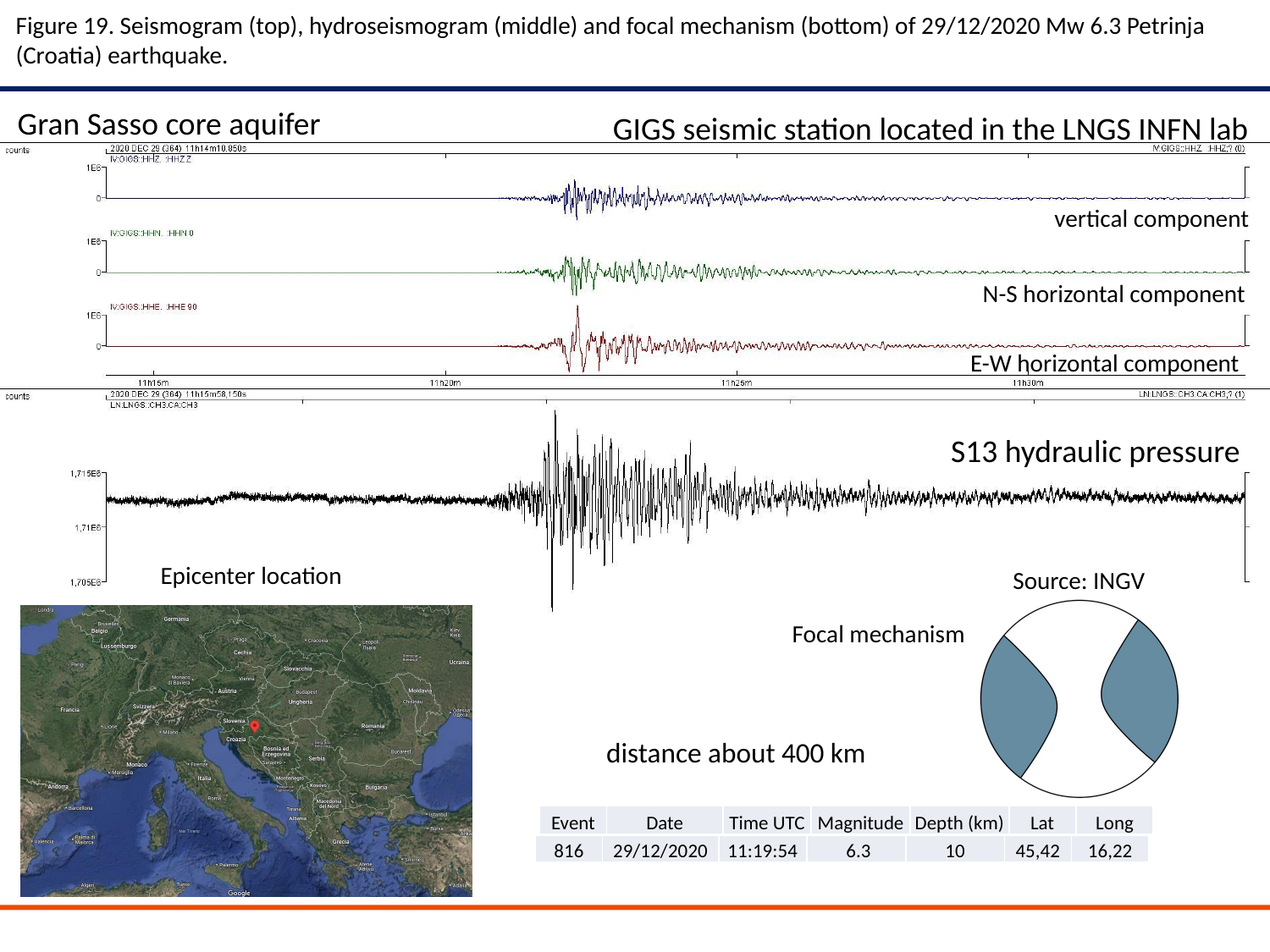

## Slide 20
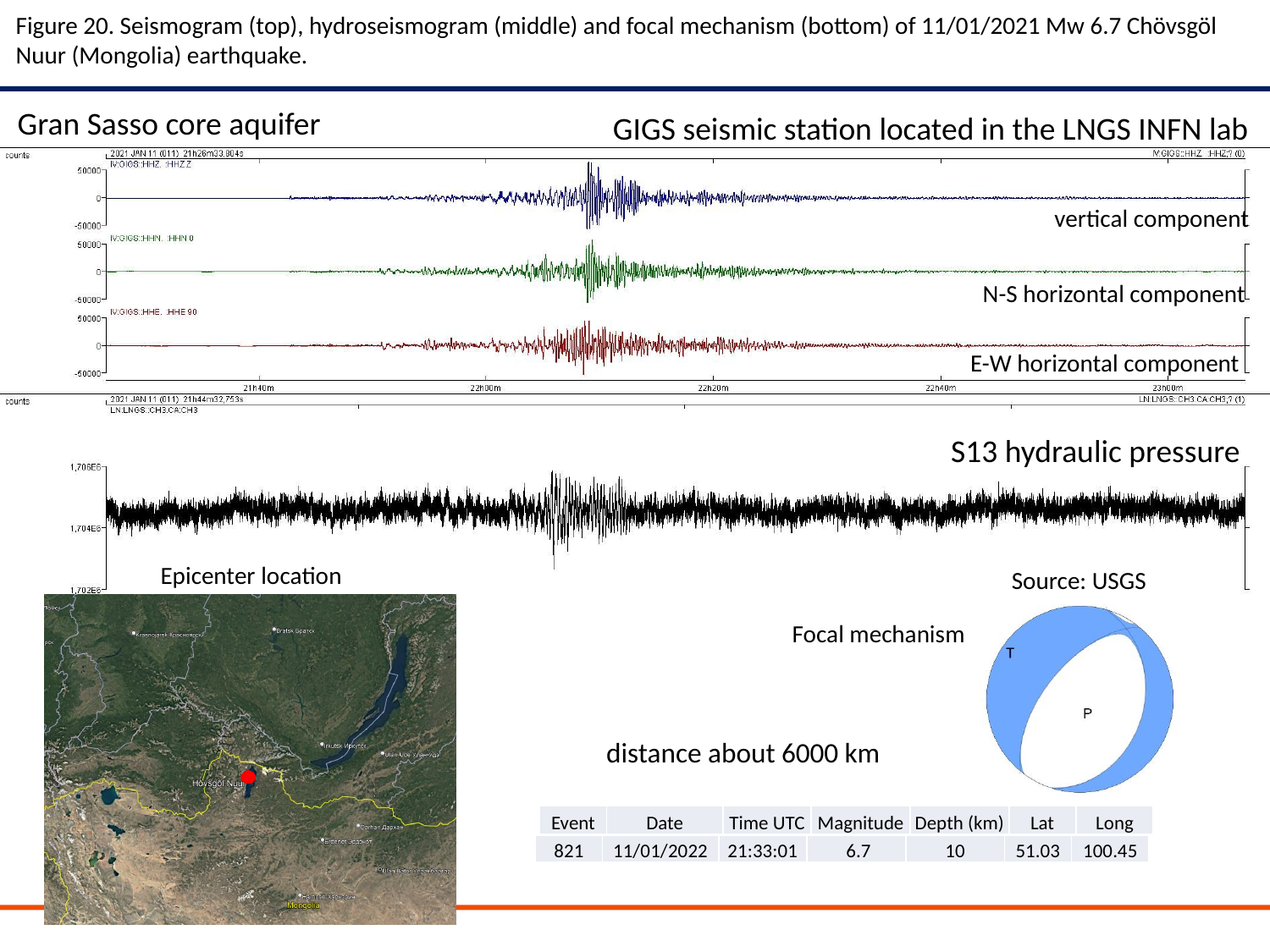

## Slide 21
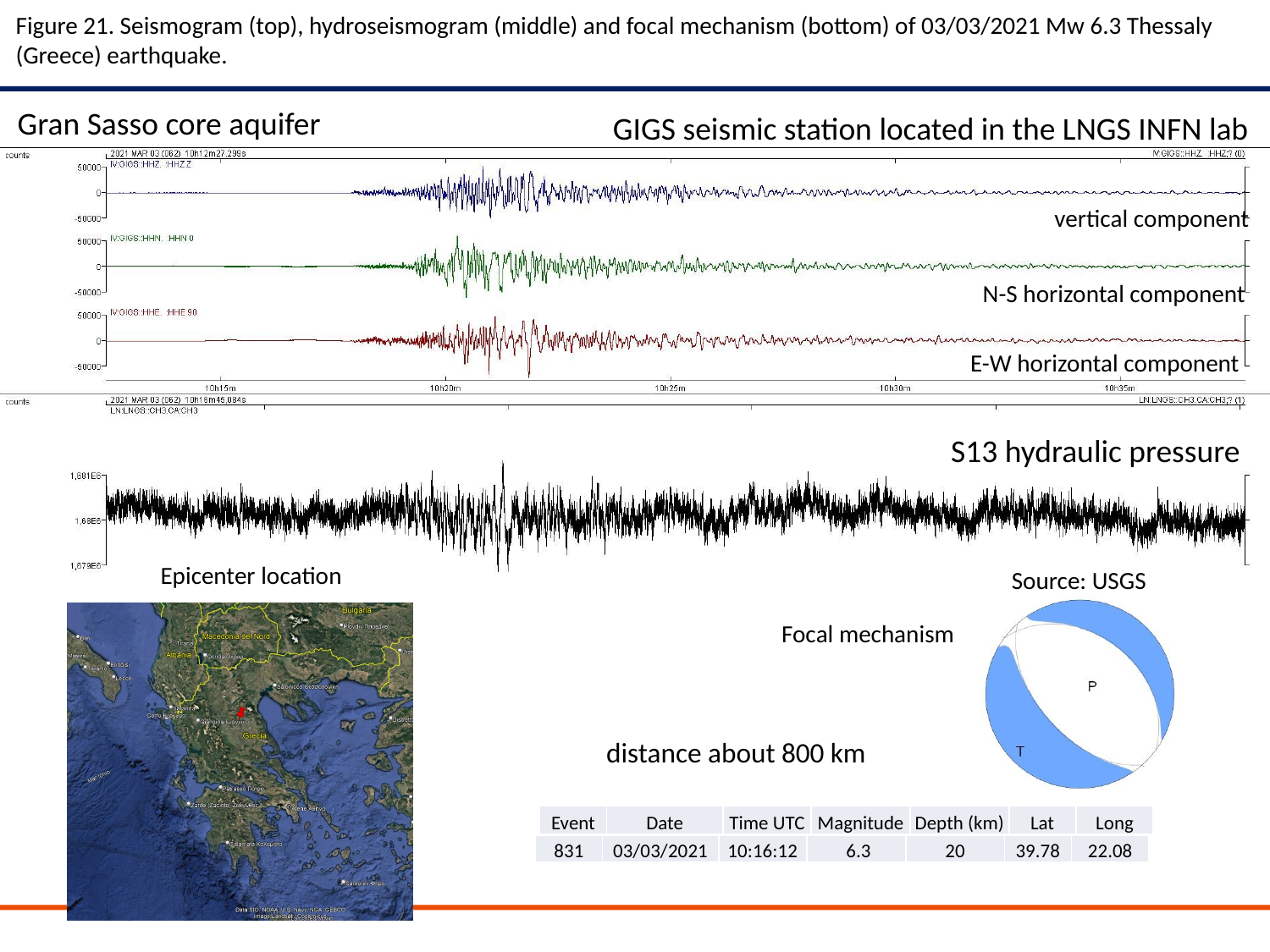

## Slide 22
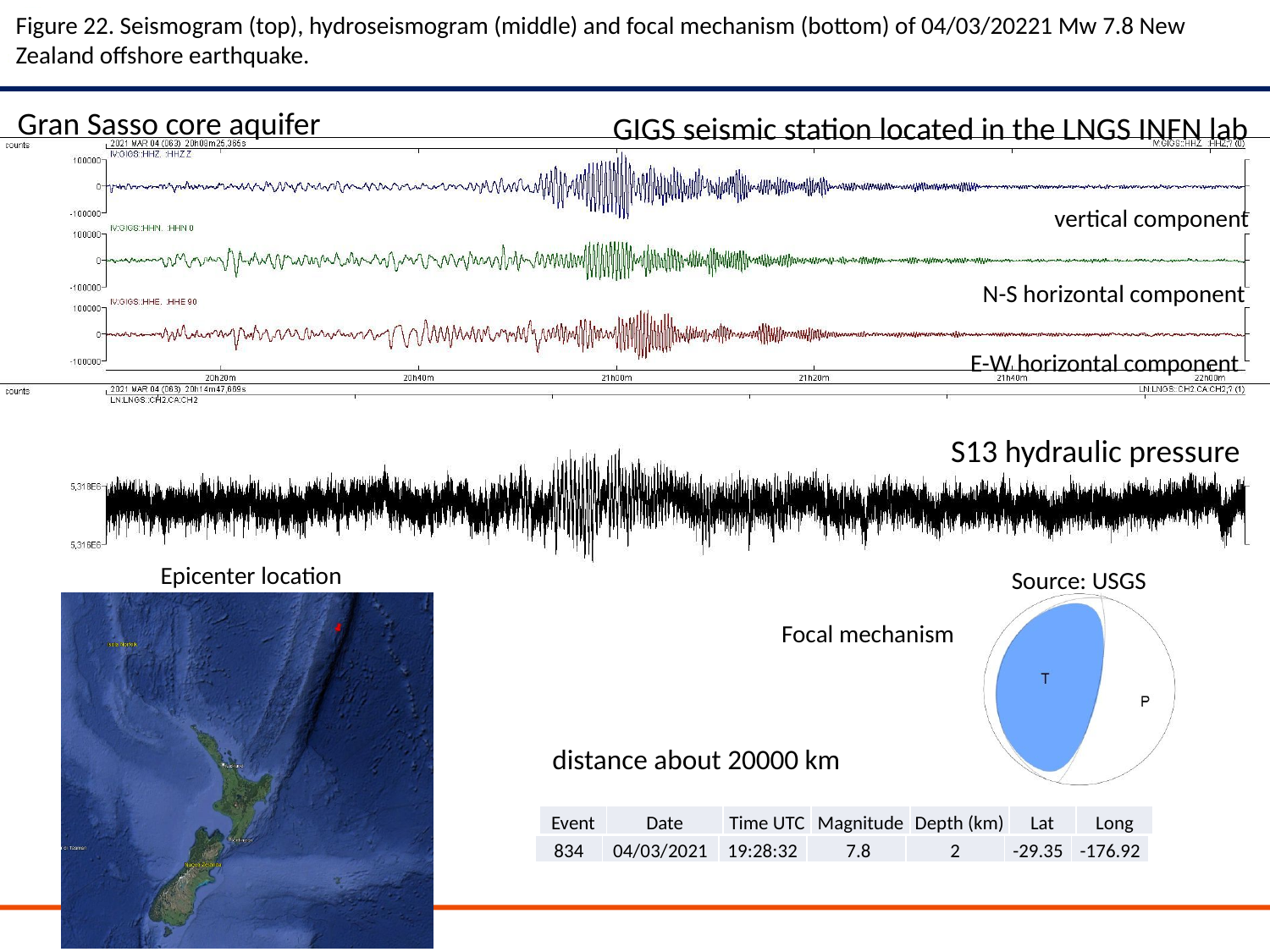

## Slide 23
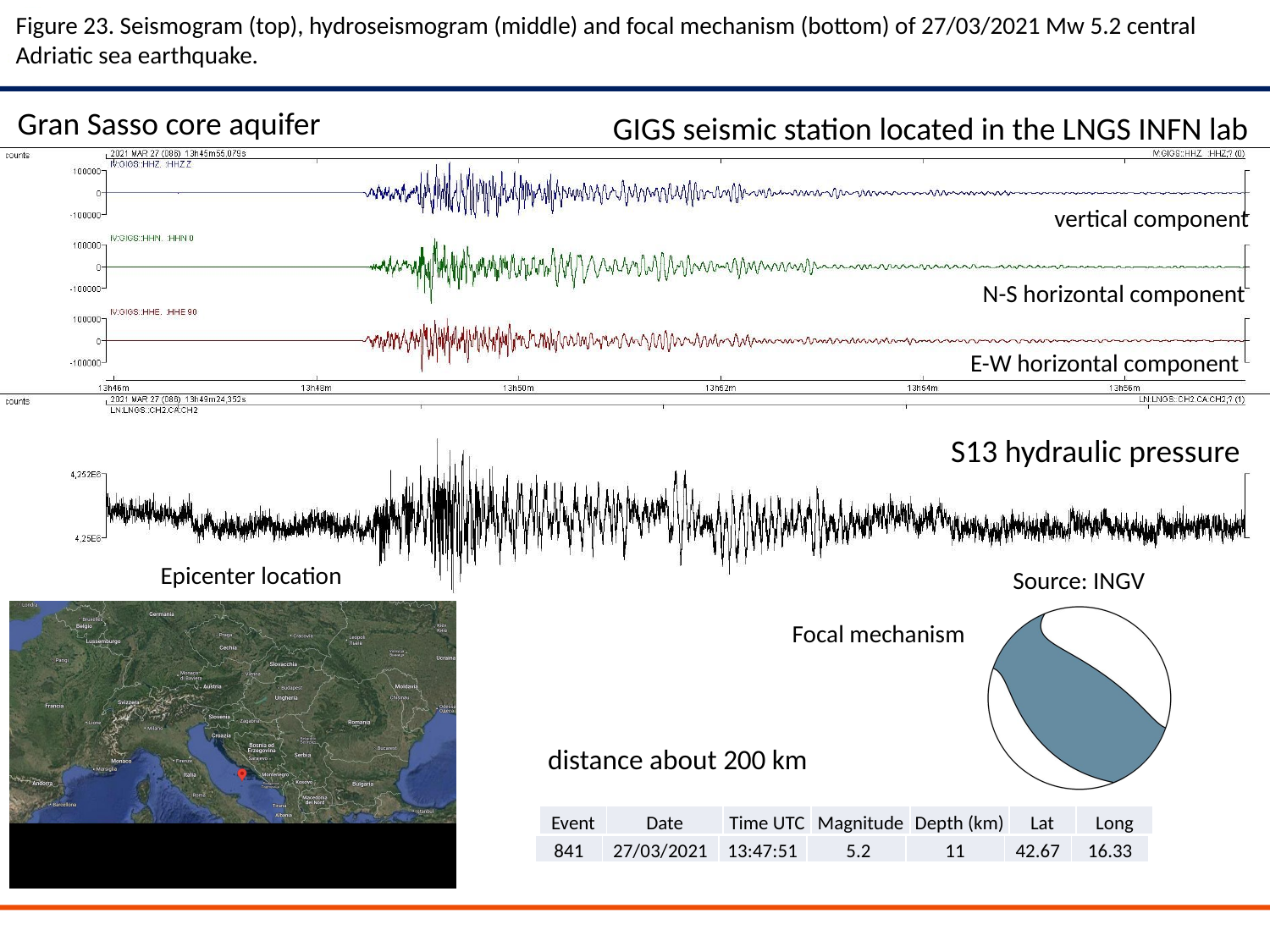

## Slide 24
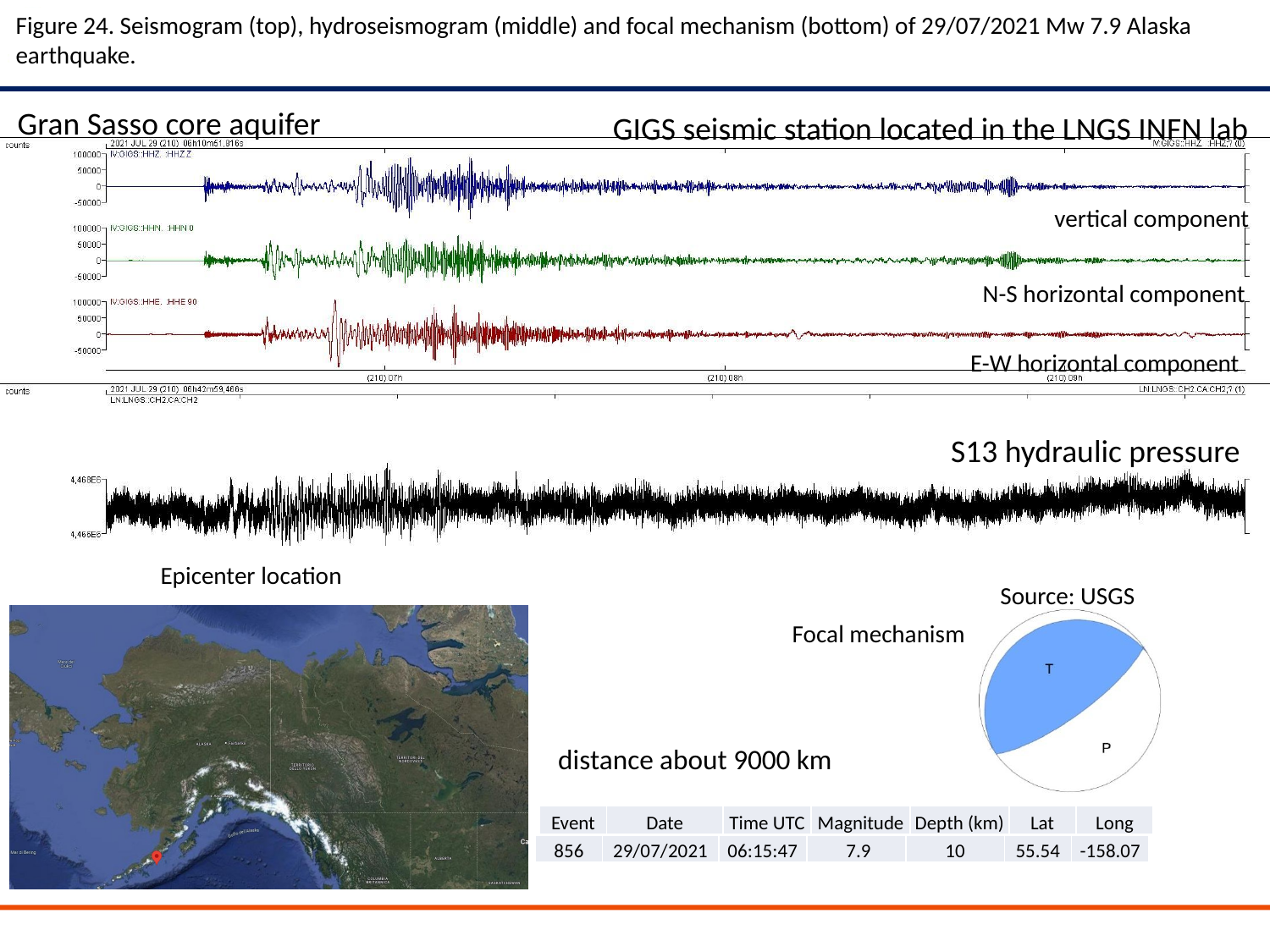

## Slide 25
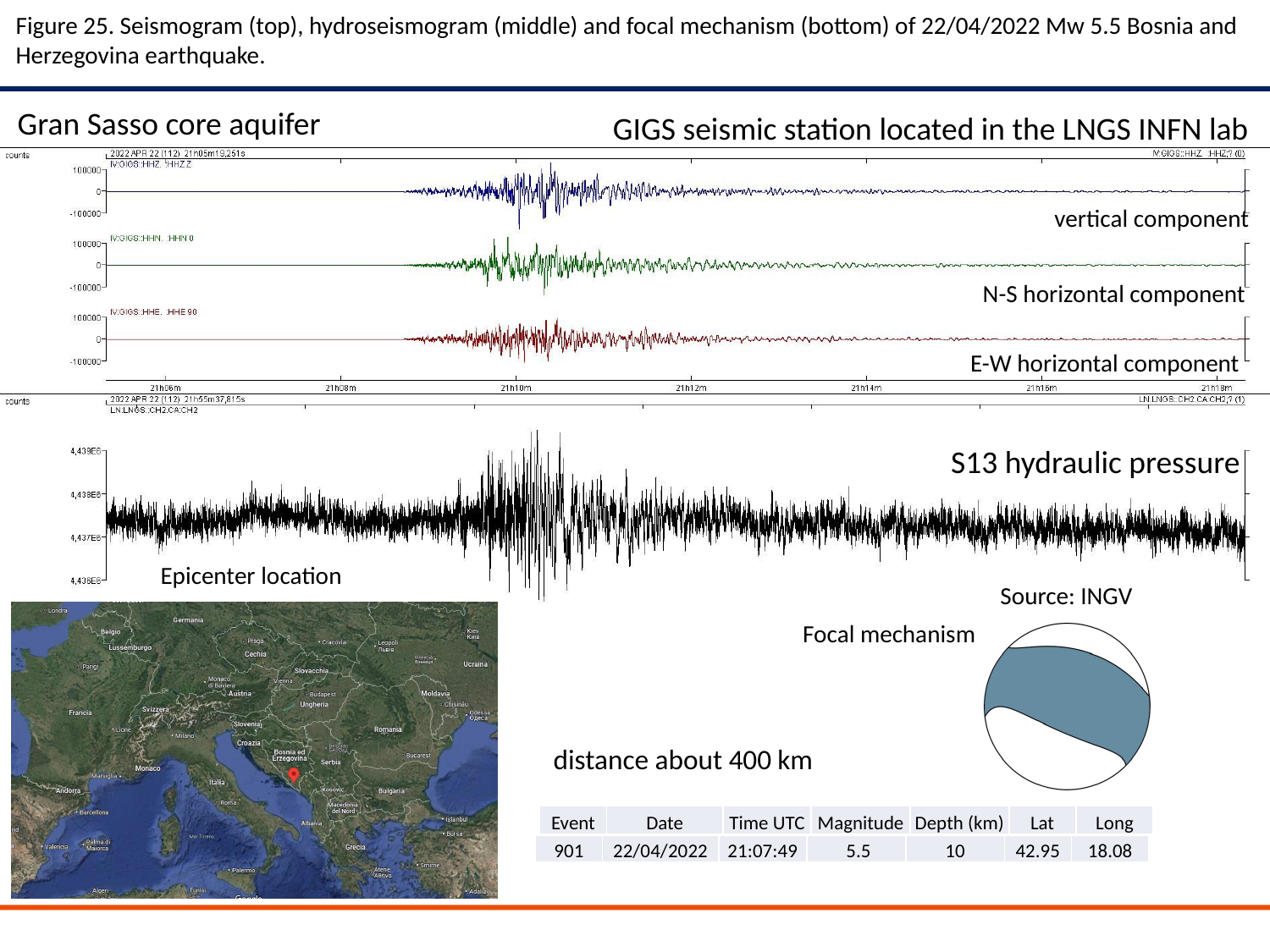

## Slide 26
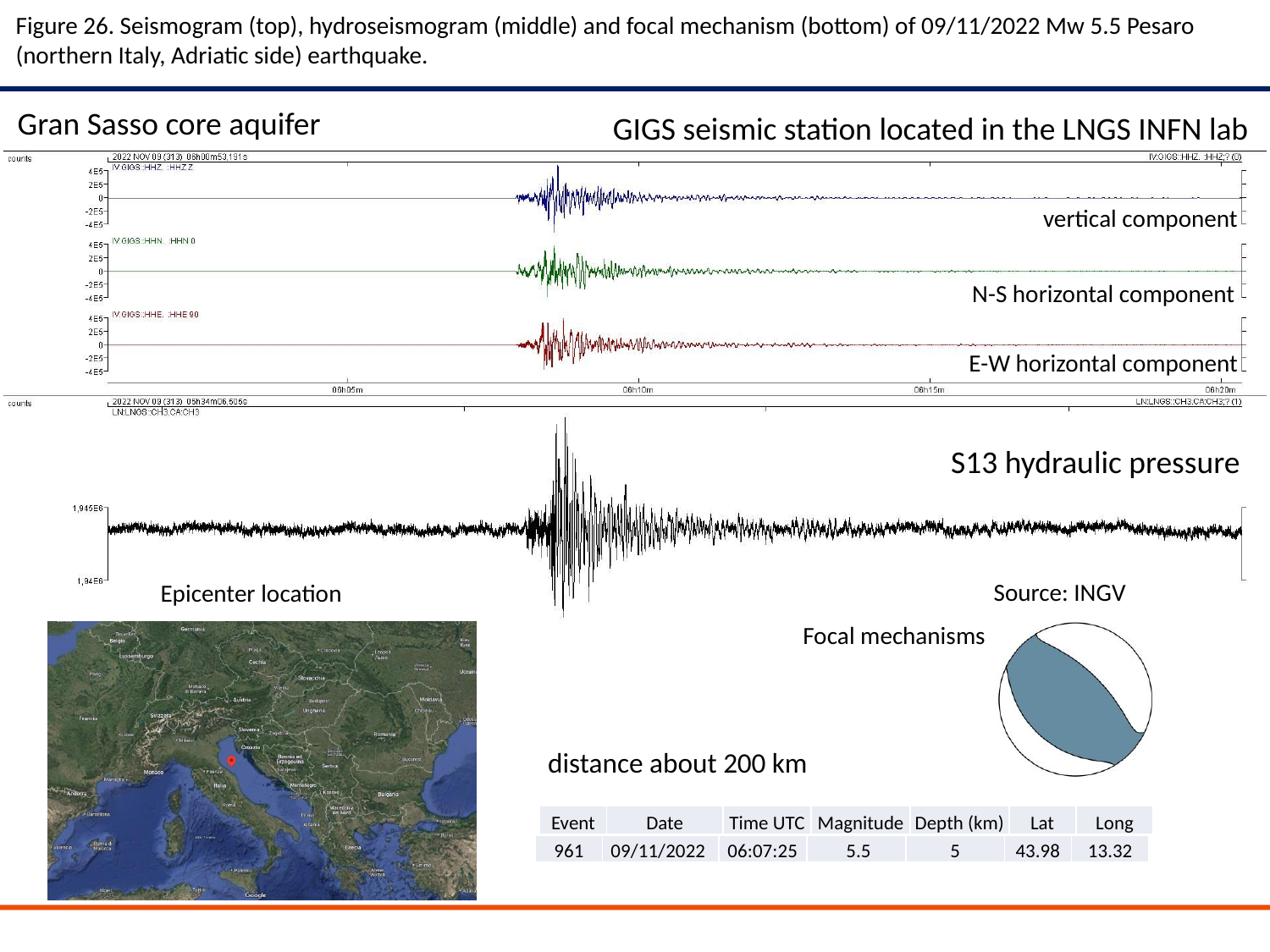

## Slide 27
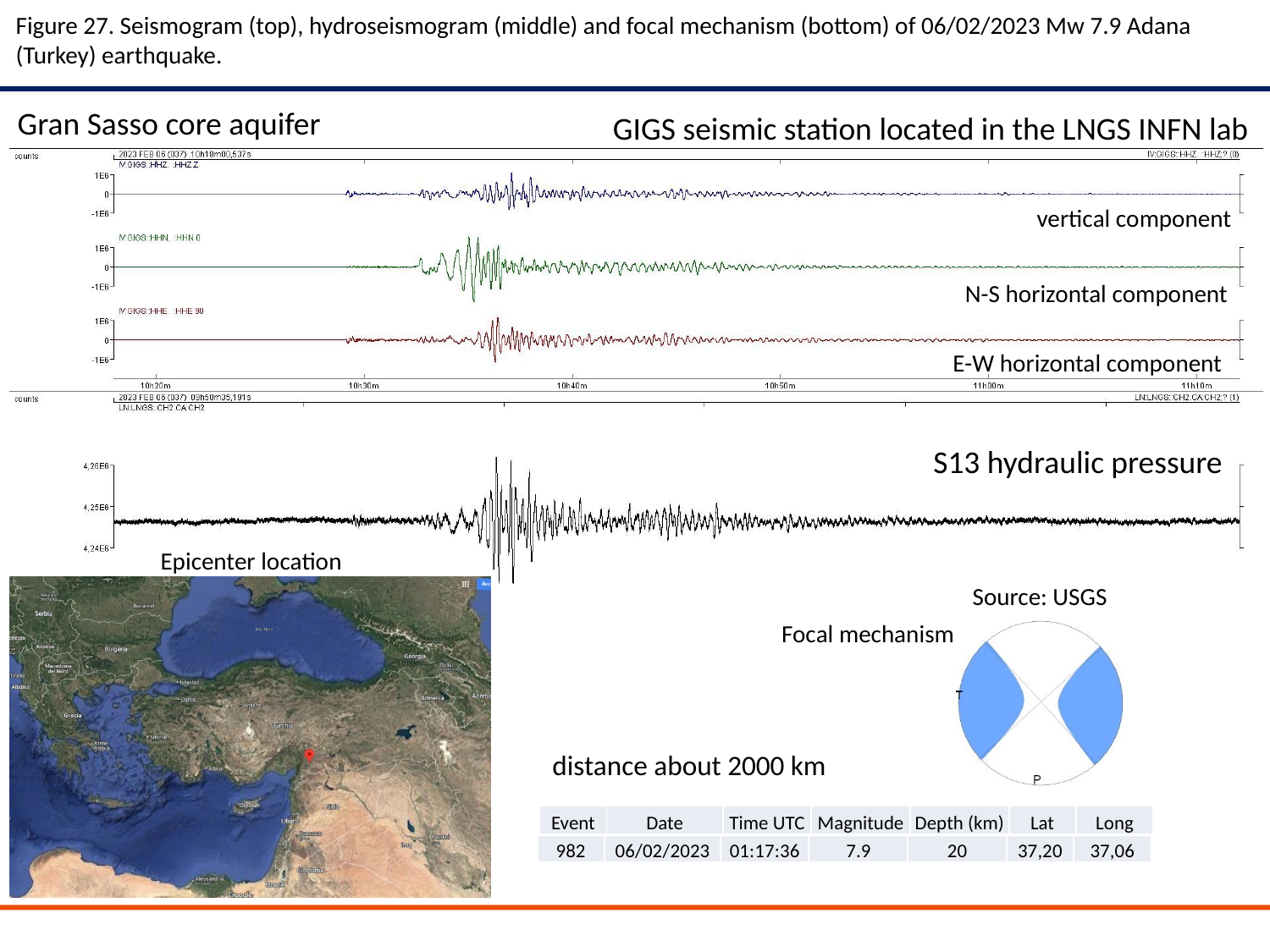

## Slide 28
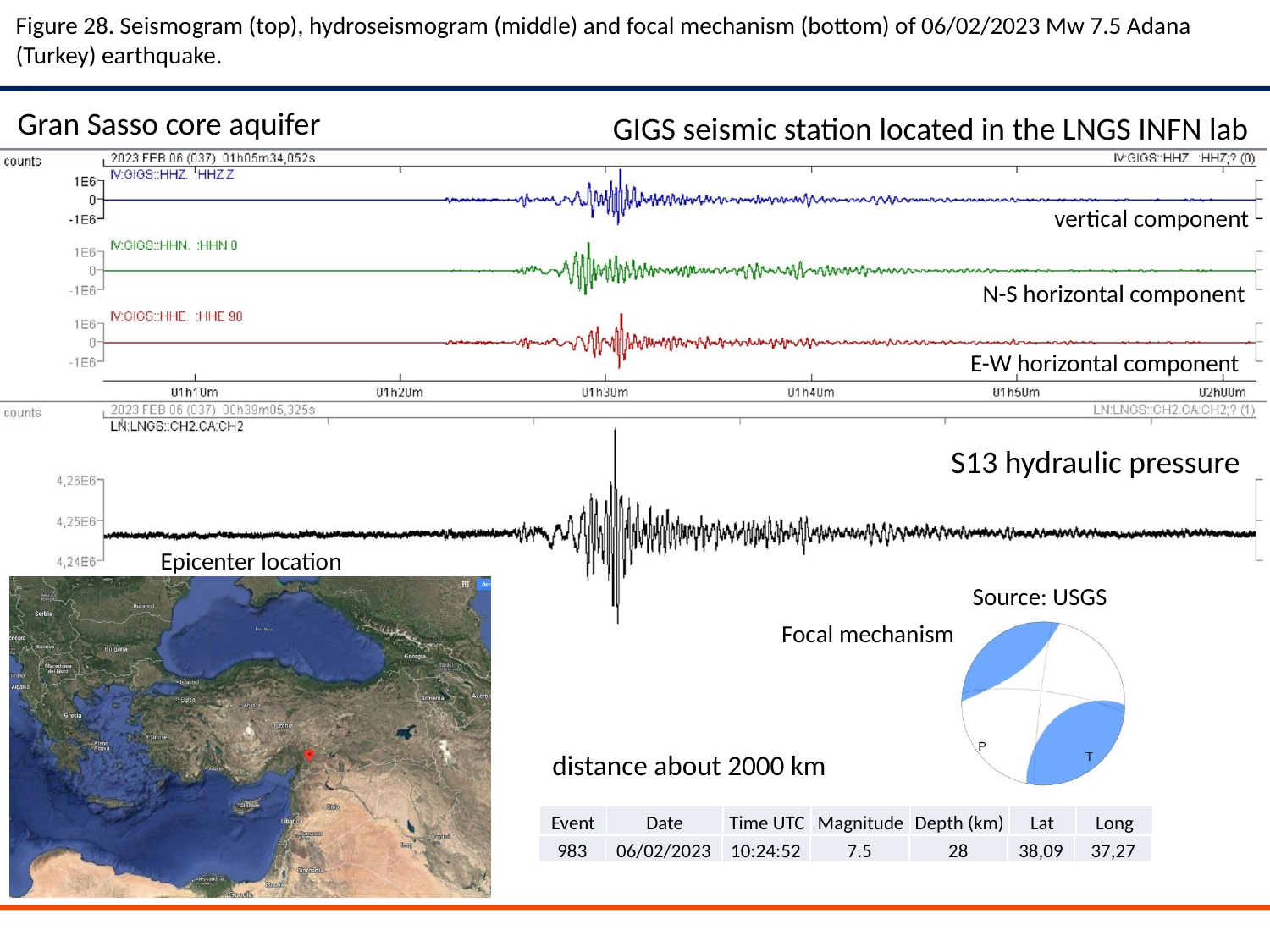

## Slide 29
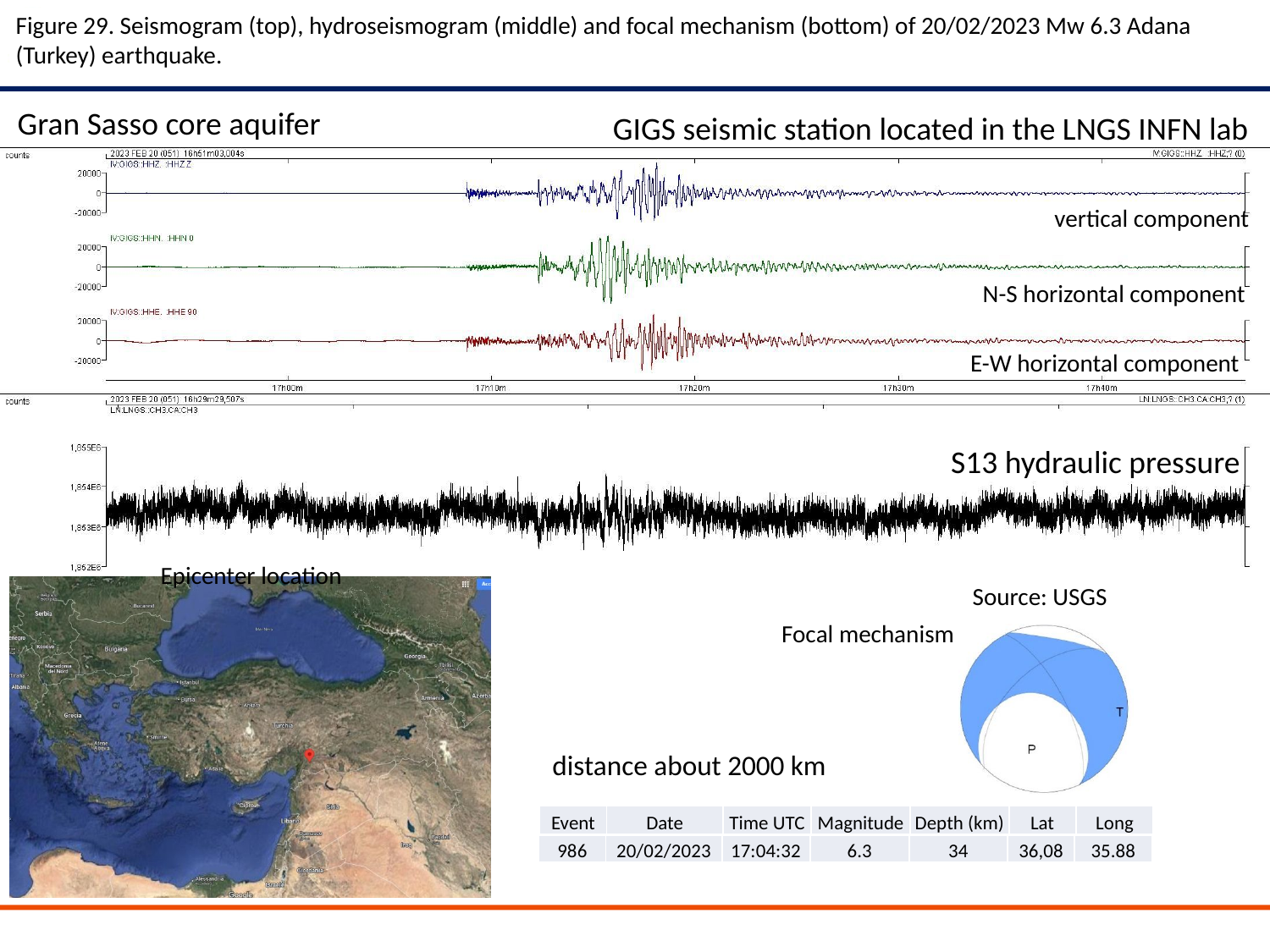

## Slide 30
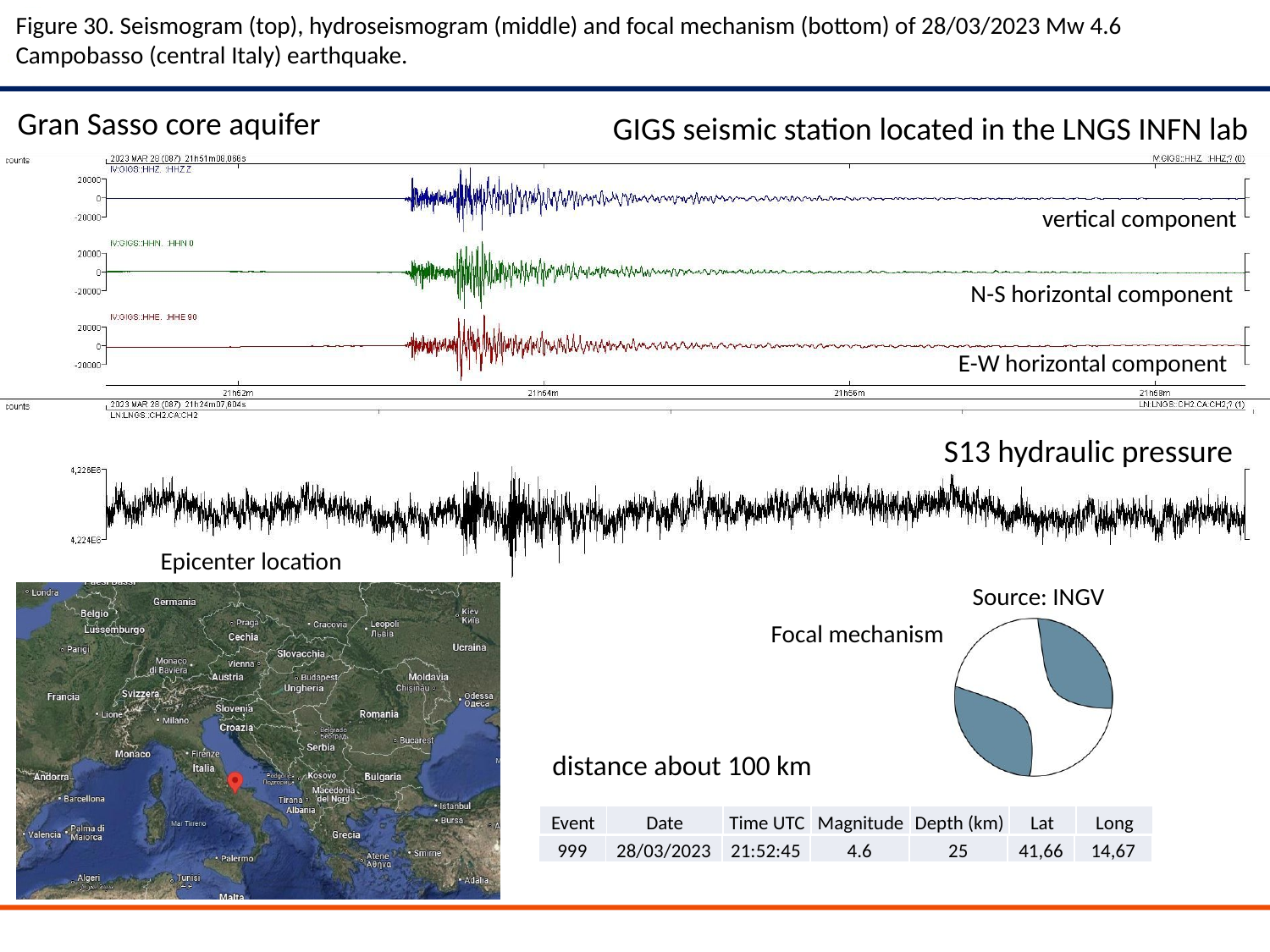

## Slide 31
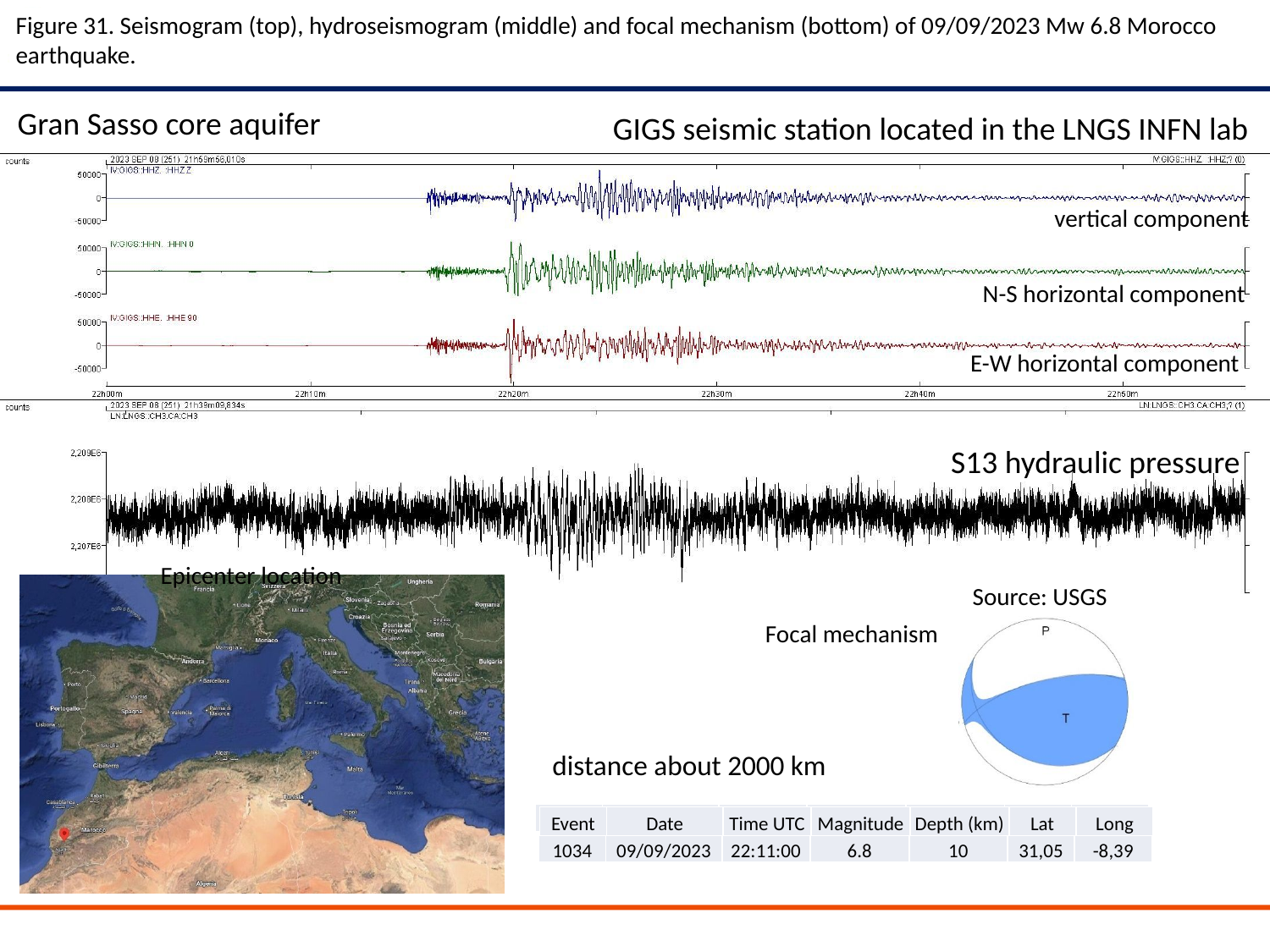

## Slide 32
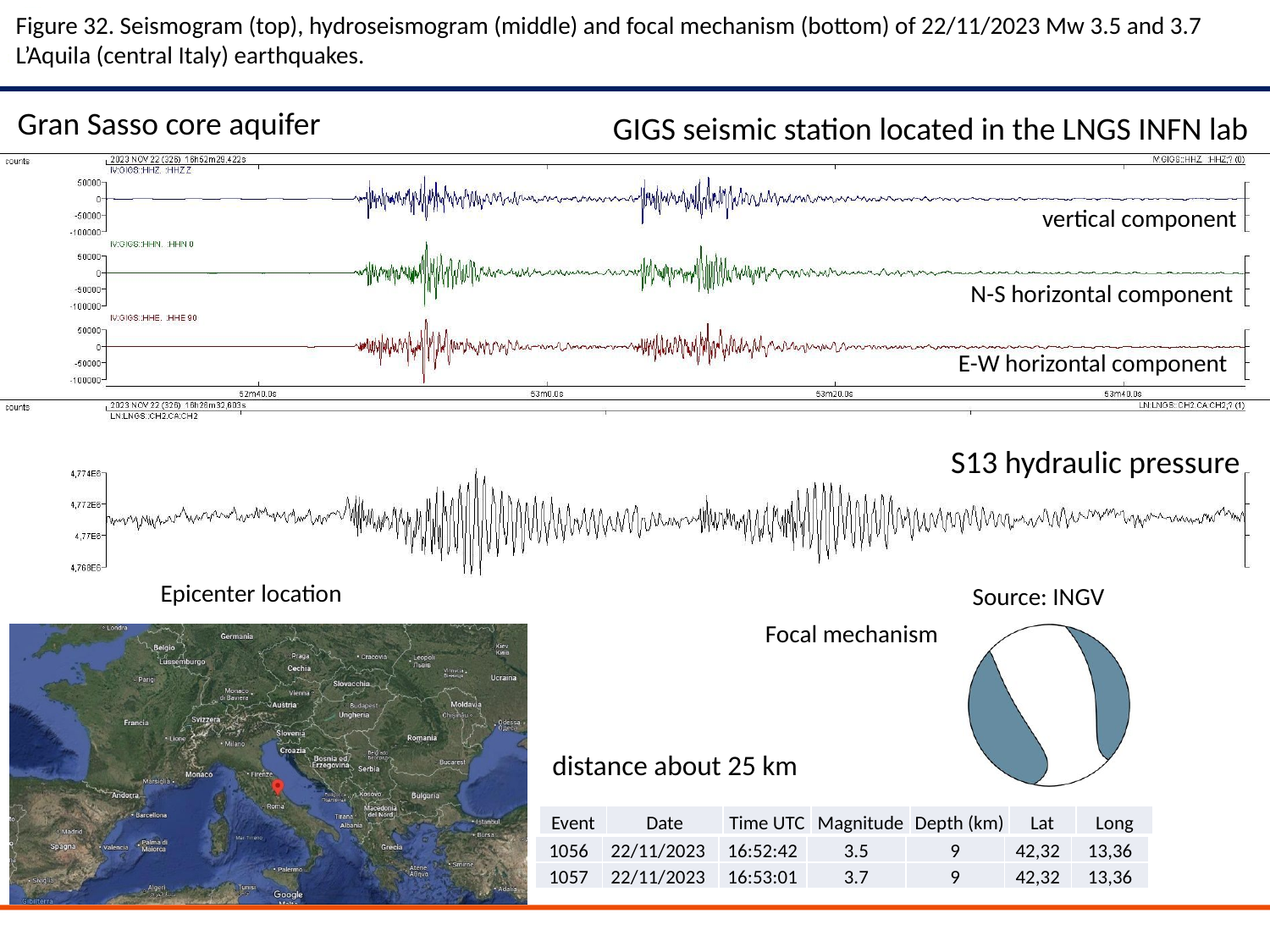

## Slide 33
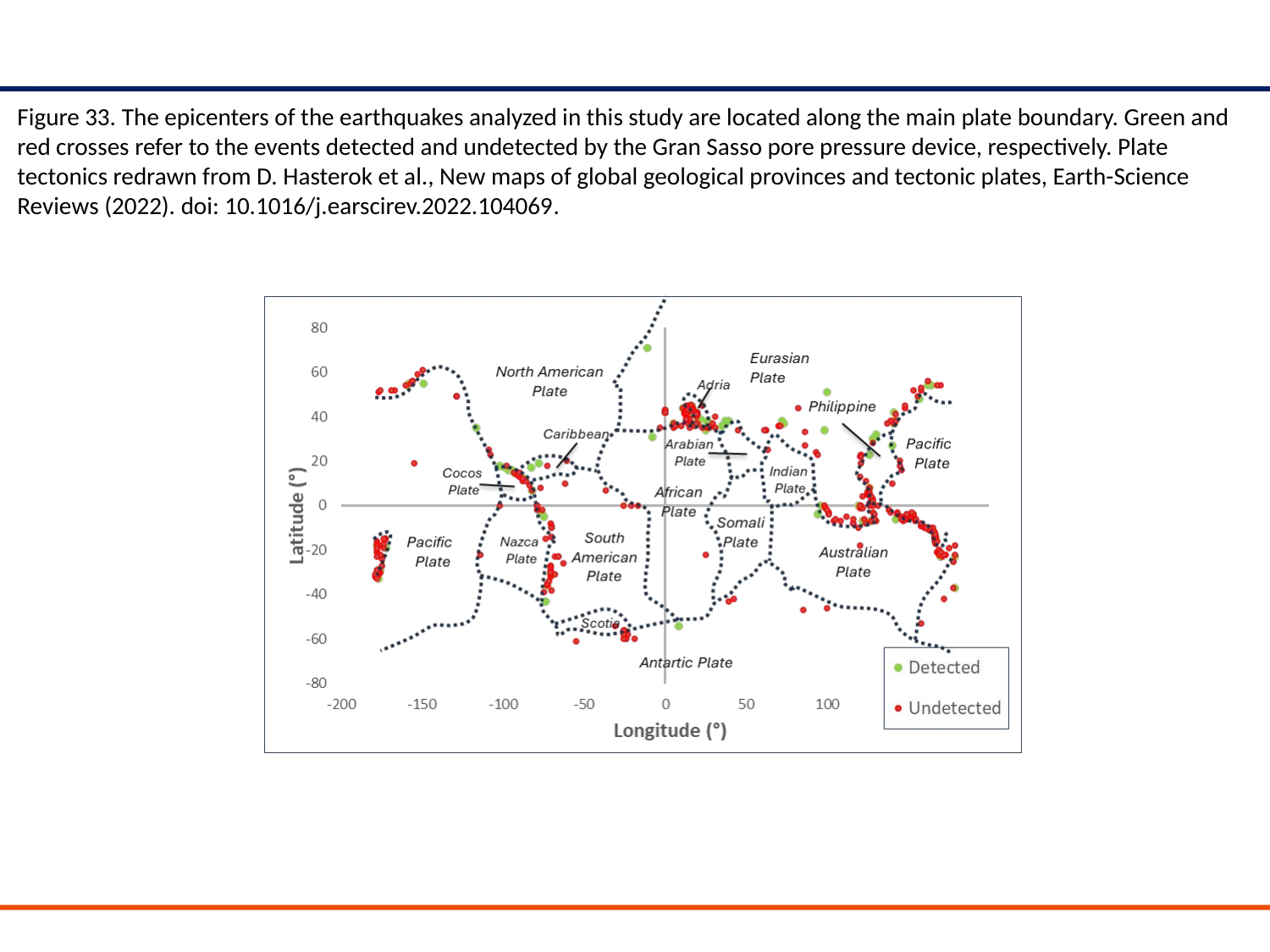

Supplement: Supplementary file 1 — Supplementary Material 1 [file 41598_2025_96113_MOESM1_ESM.pptx]
